# Uncovering extensive post-translation regulation during human cell cycle progression by integrative multi-’omics analysis

**DOI:** 10.1186/s12859-019-3150-5

**Published:** 2019-10-29

**Authors:** Gregory M. Parkes, Mahesan Niranjan

**Affiliations:** University of Southampton, University Road, Southampton, SO17 1BJ UK

**Keywords:** Cell cycle, Post-translational regulation, Predictive regression, Integrative analysis, Novelty detection

## Abstract

**Background:**

Analysis of high-throughput multi-’omics interactions across the hierarchy of expression has wide interest in making inferences with regard to biological function and biomarker discovery. Expression levels across different scales are determined by robust synthesis, regulation and degradation processes, and hence transcript (mRNA) measurements made by microarray/RNA-Seq only show modest correlation with corresponding protein levels.

**Results:**

In this work we are interested in quantitative modelling of correlation across such gene products. Building on recent work, we develop computational models spanning transcript, translation and protein levels at different stages of the *H. sapiens* cell cycle. We enhance this analysis by incorporating 25+ sequence-derived features which are likely determinants of cellular protein concentration and quantitatively select for relevant features, producing a vast dataset with thousands of genes. We reveal insights into the complex interplay between expression levels across time, using machine learning methods to highlight outliers with respect to such models as proteins associated with post-translationally regulated modes of action.

**Conclusions:**

We uncover quantitative separation between modified and degraded proteins that have roles in cell cycle regulation, chromatin remodelling and protein catabolism according to Gene Ontology; and highlight the opportunities for providing biological insights in future model systems.

## Background

The expression of proteins within a cell characterises its behaviour and function, with the mechanism of information flow from DNA template to protein degradation being an area of profound interest. This process involves multiple regulated steps from transcription, RNA processing and translation to post-translational modifications and degradation. Through estimating mRNA abundance by microarray or RNA Sequencing (RNA-Seq) in cells, we can profile gene expression assuming that mRNA and protein abundances are correlated [1, 2] and throughout literature mRNA abundance has been taken as an effective proxy for protein expression [3–5] where steady-state protein abundance has been difficult to obtain. Recent advances in RNA-Seq [6] and proteomics [7] have allowed analysis on a system-wide scale and have highlighted complex interactions between mRNA and protein previously not understood, due to transcriptomic measurements being only able to provide a distant approximation to modelling cellular behaviour, as protein abundance determines cell state. This has cast doubt on the reliability of mRNA abundance as a proxy for protein function, due to complex post-transcriptional and post-translational interactions regulating protein levels across the cell cycle.

To fully explore the regulatory interactions across the cell cycle, a multi-’omics approach must be adopted that quantifies the stages surrounding translation, in addition to mRNA and protein abundance. Indeed several studies have explored relationships to protein beyond mRNA level already, using features derived from the DNA sequence (Vogel et al. [2]), and mRNA/protein halflives (Schwanhäusser et al. [35]). Notably, these studies demonstrate the use of a multi’-omics approach as both sequence features and halflives contributed significantly to determining protein abundance. Novel system-wide translation methods have been introduced recently, that in conjunction with mRNA and protein quantification have begun to unravel the complex interplay across the ’omic scales. One of these methods is PUromycin-associated Nascent CHain Proteomics [8, 9] (PUNCH-P), which globally labels newly synthesized proteins and estimates quantity using mass-spectrometric (MS) analysis, leading to a ’snapshot’ of the translatome. Zur et al. [10] describes experimental comparisons between PUNCH-P and the more familiar Ribosome Profiling (Ribo-Seq) technique which arrests translation and sequences protected mRNA fragments. Using both mRNA and translation abundance, this provides a basis for powerful statistical analysis for protein abundance prediction, by accounting for post-transcriptional modifications. Previous studies [8] have explored the interplay between mRNA, translation and protein at different time steps within the cell cycle, but prediction of protein abundance using multi-’omics expression data across the cell cycle has not been explored in significant detail.

Various authors have considered probabilistic approaches such as Bayesian modelling [11] and coupled-mixture modelling [12] to investigate the relationship between transcriptome and proteome measurements. However we integrate novelty detection using outliers instead as done previously [13]; by building a powerful statistical model where inputs are carefully selected, examples of predicted outputs where accuracy is weak are informative. Here, we extend work previously done [8] by integrating expression data from multiple stages in the protein development pathway with information contained within the known primary DNA/RNA/amino-acid sequence of said related expression data. By selecting features that occur/describe events before the protein is created, we hypothesize that any proteins with a large predicted-to-actual protein abundance ratio, will bear significant post-translational modification and/or degradation functionality, as first proposed by [13]. Tuller et al.[15, 25] and Gunawardana et al. [13, 14] have developed data-driven models to predict protein abundance in *S. cerevisiae* and prokaryotic organisms. Using various feature selection methods such as Greedy Forward/Backward and *l*_1_-norm sparsity-induced regularization (LASSO), previous studies have identified sequence-derived features such as tRNA Adaptation (tAI), Codon Adaptation (CAI) and evolutionary rate (ER) [2, 13, 15] among others as relevant predictive features, in conjunction with mRNA expression levels. Indeed, some of these models have been relatively successful in achieving very-high correlations between predicted and actual protein abundance (*R*^2^=0.76,0.86).

Post-translational modifications (PTMs) following protein biosynthesis are fairly common to many proteins, in particular phosphorylation; and are known to promote an array of functions including cell signalling, protein folding and ubiquitination [16]. In particular, PTMs are known triggers of proteolytic degradation either through cause or by consequence of oxidative stress, due to the modification of specific amino-acid sites to alter the protein’s tertiary/quaternary structure [17, 18]. This can lead to compromised in *vivo* protein stability at a local level (such as protein methylation) or at the C/N-terminal regions. A number of PTMs involve covalent bonding with members of the ubiquitin family through ubiquitylation/sumoylation. In addition to this, phosphorylation (the most frequent PTM) has been shown to bear complex cross-talk with ubiquitin-like factors [19].

The focus of this work is the merging of high-quality multi-’omics measurements with rigorous machine-learning technique for dynamic-system/time-series protein prediction. Combining this approach with first-principle novelty detection theory leads to a powerful iterative approach to understanding outlier effects in proteins. We have one of the highest correlations (*R*^2^=0.64) across multiple timesteps for human proteome prediction (most studies do not explore across time). We assembled a modest dataset with over 3500 rows with no missing data, across 30 different features; which is accessible for public use and will provide a benchmark for future human proteome prediction studies. In addition, we have unpacked some of the complex separation between post-translational modification and degradation signalling in proteins difficult to predict that reveal insight into key mechanisms across the cell cycle.

## Results

To develop a protein abundance predictor across the cell cycle, we take data collected from Aviner et al. [8] containing triplicative measurements of transcriptome (microarray), translatome (PUNCH-P) [9] and proteome (Mass Spectrometry; MS) at stage G1 growth phase (2h), S phase (8h) and G2/M phase (14h) from synchronized HeLa cells used in the same study [20]. This provides a base set of multi-’omics measurements for 6785 transcript levels, with around 4700 non-missing protein/translation abundances. In order to allow comparison across the gene product hierarchy, mRNA and protein were experimentally normalized by analyzing the same quantities of biological material at each phase, and translation measurements were normalized by the number of translating ribosomes at each phase.

### Translation rates have significantly higher predictive power for protein abundance than transcript levels alone

**Fig. 1:**
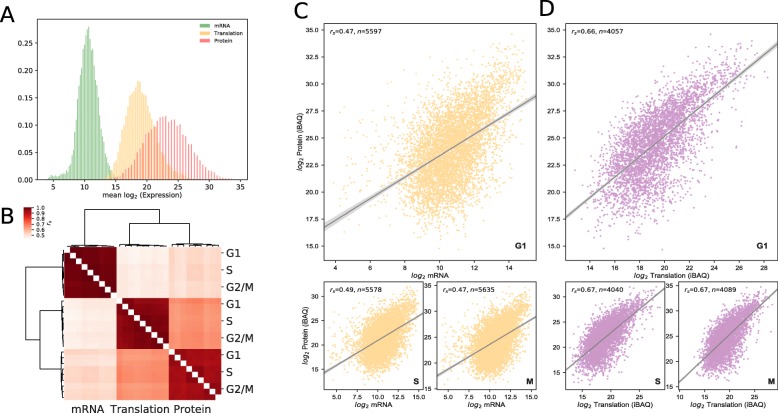
Distributions and Correlations in transcript, translation and protein levels. Published data from [8], shown for completeness. **a** Histogram distributions of mean *log*_2_ mRNA (microarray), translation (PUNCH-P) and protein (MS) levels for combined cell cycle time points. **b** Cluster mapping of Spearman-rank (*r*_*s*_) correlation matrix for mean *log*_2_ mRNA, translation and protein levels at G1, S and G2/M cell cycle phases. **c-d** Scatterplots of mean *log*_2_ mRNA versus protein (**c**) and translation versus protein (**d**) for each cell cycle phase. M abbrievated represents G2/M phase. *n* refers to the number of samples

Since multiple copies of a protein are often produced from a single mRNA strand, we expect translation/protein abundance and variance to be greater than mRNA levels. Indeed, we see translation and protein levels to be several orders of magnitude larger than mRNA (Fig. 1a), with a larger span indicative of higher variance. Hierarchical clustering of Spearman-rank correlations between triplicative measurements of gene products (Fig. 1b) shows high intra-correlations across the ’omic scale, with translation clustering closer to protein than mRNA. This demonstrates the apparent invariance across the three cell cycle phases in preference to differences between gene products, with mild correlation between transcript and protein levels (*r*_*s*_=0.47-0.49) across all phases, as demonstrated in the original work and by other authors for mammalian cells [3, 8, 21]. Correlations of translation against protein are significantly higher (*r*_*s*_=0.66-0.67) at all time points, which is not due to the technical similarity in measurement technique. This is likely due to translation level accounting for robust post-transcriptional mechanisms applied across the transcriptome, such as alternative splicing and mRNA degradation [22]. Visualisation of correlation (Fig. 1c,d) shows an consistent left skew in mRNA versus protein plots, contributing to a reduction in positive correlation compared to translation. To see whether this artefact is due to the reduction in sample size *n* alone (5500 to 4000), we separated mRNA measurements by whether they had missing translation level data or not, and calculated *r*_*s*_ for each sub sample (Additional file 1). We do see a drop in correlation (*r*_*s*_=0.23-0.24) in samples with missing translation data versus samples with data (maintained at stated level), this may be due to experimental issues with measuring low levels of translation in these genes, and since protein stability can be inferred from translation level (as shown previously [8]), these proteins may not be sufficiently steady-state. Alternatively, due to the low resolution of only having three time points (G1, S and G2/M), these labile proteins may be below the detection threshold at the time of measurement.

Further to this, we developed a naive protein abundance predictor with a bias term using just mRNA and translation levels as input to a linear model (Additional file 2). This illustrates that once translation is known, mRNA levels become mostly redundant in protein abundance prediction as there is a negligible increase in correlation. Therefore, we extracted new sequence-based and frequency-based features known before protein synthesis to use as inputs for a machine learning predictor model. Our downstream analysis develops this to expand the original dataset to discover new insights across the cell cycle.

### Sequence-based features cumulatively improve prediction, but individually correlate weakly

**Fig. 2:**
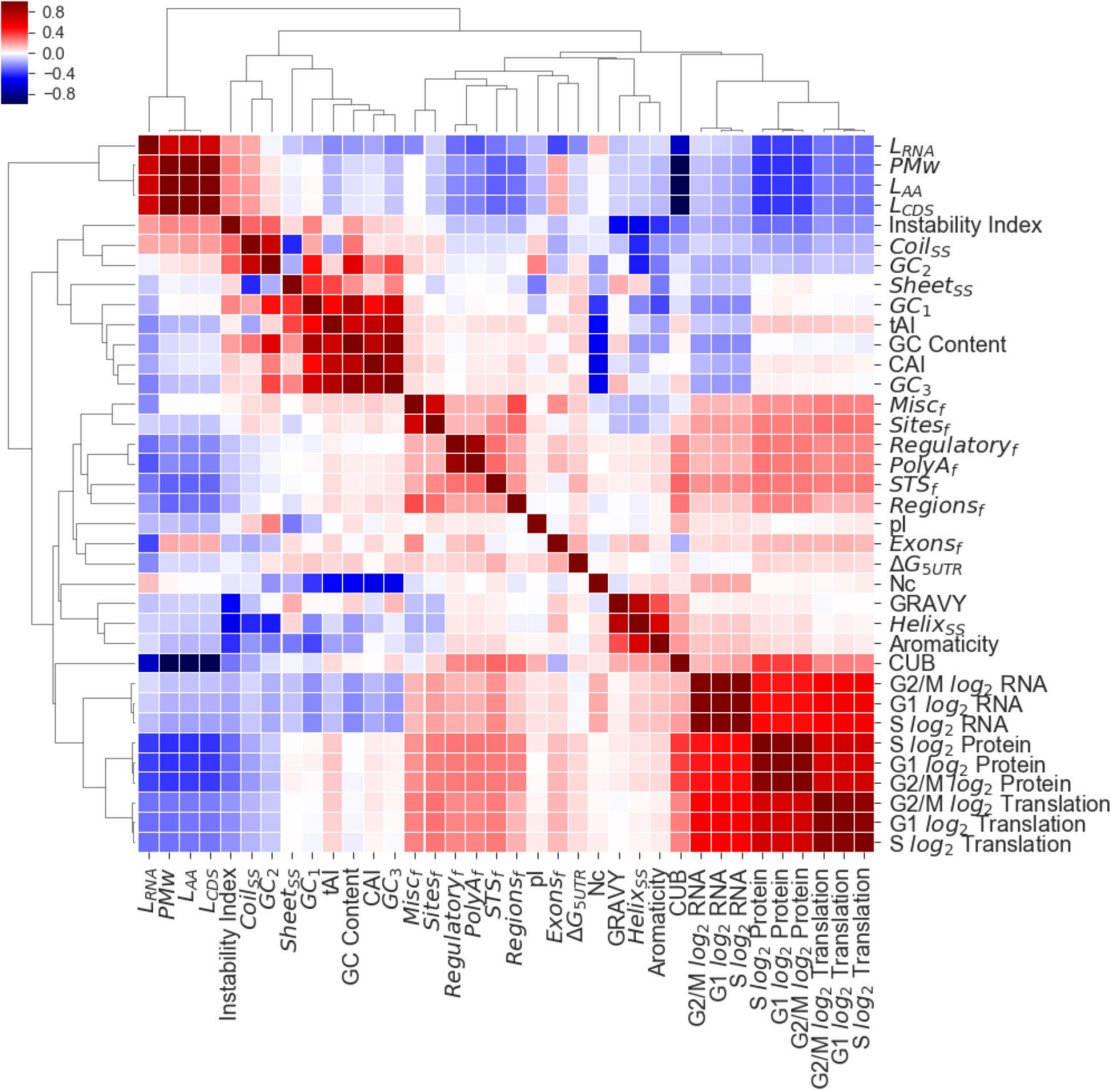
Insightful inter-correlations across sequence-derived and gene expression data. Hierarchical clustering of pairwise Spearman-rank (*r*_*s*_) correlation matrix for all input features including target (protein). See Additional file 8 for abbrievated labels. Intensity scale differs from Fig. 1b in bottom-right corner visually but numerically exact

We mined for features primarily from curated RefSeq mRNA transcripts and associated amino-acid sequences (beginning with NM_ or NP_) from the NCBI Entrez database [23] using HGNC gene names [24]. Sequence-derived features were extracted from the underlying mRNA or coding sequence (CDS), in addition to frequency-based features that are identified in the Genbank feature table, and are described here (Additional file 8). The resulting dataset is fully available to all readers in (Additional file 10). Next, we explore pairwise correlations between all the features, as well as their correlations to the target protein concentrations as a clustered intensity plot (Fig. 2), with translation, mRNA levels, sequence-length/protein molecular weight (PMw) and CUB with the largest absolute Spearman-rank correlations to protein level (*r*_*s*_=0.66, 0.47, −0.4, 0.37 respectively). Interestingly the negative correlation between Length/PMw to protein level would suggest that larger proteins are more likely to have lower abundance across all phases. Indeed we would expect enzymatic proteins, known to be smaller; to be higher in abundance than larger proteins which predominantly involve structural interactions.

Further to this, the comparatively small correlation of tAI and CAI with respect to protein with regards to previous authors [2, 13, 25] may be due to differences in gene regulation complexity between humans/yeast. However, the correlation matrix does not inform on how features will cumulatively interact with each other in any subsequent models, therefore making it difficult to identify redundant features. To examine this effect, we performed principle component analysis (PCA) on the input matrix (i.e all the features minus protein) to see how much explained variance can be in the largest eigenvalues (Additional file 3). Whilst there is noticeable dominance within the first six principle components, there is not a clear exponential decay in feature importance, indicating that there are small, cumulative factors at play in these features that may contribute independently useful information. In addition, the assumption of linearity required for PCA transformation use may not hold true in the biological system due to complex interactions between mRNA and protein in vivo. Further to this, we examined the scatterplots from t-distributed stochastic neighbor embedding (t-SNE) and observed uniform scattering/little structure in reduced dimensions. Due to these reasons, we used feature selection instead of PCA in downstream analyses.

### Differences across the cell cycle begin to emerge when selecting important features

To examine the potential of different computational methods on this dataset, we performed 10-fold cross validation on different regressors across all phases (Additional file 3), with gradient-boosted regression trees (GBRT) consistently providing marginally higher accuracy on out-of-sample data (*R*^2^= 0.64±0.06) than other methods, and performing significantly better than using just mRNA and translation as inputs (*R*^2^= 0.49±0.02). We note that GBRT is non-linear in it’s approach, and fairly robust to overfitting due to averaging over base tree estimators. It is interesting to observe the surprisingly good performance of simpler algorithms like Ordinary Least Squares (OLS) still achieving reasonable out-of-sample accuracies (*R*^2^= 0.61±0.06), confirming the robustness of the dataset and highlighting it’s case for continued use in future studies in protein prediction. Indeed, both Gunawardana [13] and Tuller [15] found non-linear models (such as neural networks) brought little benefit and even reduced correlations. In addition, both Gunawardana and Tuller got larger correlations from linear models (*R*^2^=0.86,0.76 respectively) but both developed models for steady-state yeast, not dynamic human cells. We do however observe marginal non-linearity in scatterplots (Fig. 1c,d) at extrema thus supporting the use of a non-linear method. However in the interests of reducing overestimation from correlations within related features, we deployed three different methods of feature selection as no method is known to be optimum:

1. Recursive Feature Elimination (RFE)
2. *L*_1_ sparsity-inducing regularization (LASSO)
3. Selecting *k*-Best (ANOVA)

**Fig. 3:**
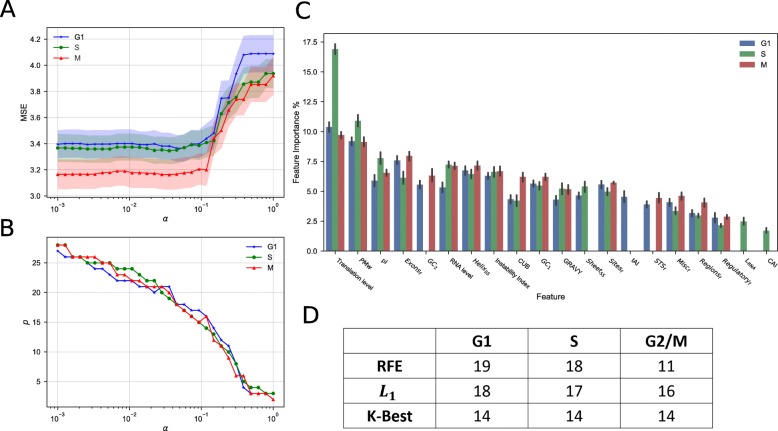
Impact of *L*_1_-regularization on reduced feature sets. **a-b** Line-plots representing parameter tuning of regularizing term *α* against the mean-squared error (**a**, MSE) and the number of features remaining (**b**, *p*), across all cell cycle phases, where *α*∈[10^−3^,1]. Error bars indicate ±SD with 10-fold CV. **c** Bar plot representation of model coefficients (as importance) for each feature from the Gradient-boosted regression tree (GBRT), using optimized *α*, for each cell cycle phase. Error bars indicate ±SD with 10-fold CV. **d** Table of number of selected features per method, per cell cycle phase

For inducing an appropriate amount of sparsity into the input matrix using *L*_1_ regularization, selecting the regularizing term *α* is crucial. We observe a dramatic increase in mean-squared error (MSE) rate with *α*>0.1 (Fig. 3a) across all cell cycle phases, while the number of features remaining *p* falls linearly as *α* increases (Fig. 3b), showing strong redundancy with at least half (14) of all features. Using the optimized *α*, we created a GBRT model (with 10-fold cross validation (CV)) using the regularized feature matrix generated from CV Lasso models, and describe the model coefficients as feature importances (Fig. 3c). Unsurprisingly, translation level dominates as the most important feature across all phases, but the remaining features mostly appear to have similar importance (5-8%), with amino-acid derived features such as PMw and pI, on average, performing better than traditionally used mRNA-based metrics like tAI or CAI. All 3 of the feature selectors reduced the most number of features from G2/M phase compared to G1 (Fig. 3d), which may suggest G1 and S proteins may be affected by post-translational regulations. To view the details of the feature selection procedure, see Additional file 11.

Here we see divergence from work done on other model organisms (such as yeast and *E. coli*), which have shown strong correlation contributions from codon bias metrics like tAI and CAI [13, 15]. We suspect this is due to the increased presence of post-translational modifications (PTMs) within higher-order organisms like *H. sapiens*, causing fluctuations on protein abundance that act as noise to the correlation with these mRNA-based metrics. It’s also a possible factor that tAI/CAI information value is simply absorbed into translation/PUNCH-P measurements rendering their contributions somewhat smaller when combined with translation. We note the increased skew of feature importances within S phase (significantly larger translation, PMw, pI), possibly indicating that these features are more active in predicting DNA replication/repair mechanisms associated with this phase. In the original work, Aviner et al. [8] also explored S phase regulation in more detail in their further analysis in relation to fold changes, therefore complexities in S phase may indicate more frequent post-translational modifications. However exploring the importance of each feature only begins to provide biological interpretation into the complex interplay between features - our primary interest is novelty detection in outliers with respect to a predictive model.

### Overestimation in majority of protein outliers indicates post translational modification or degradation

**Fig. 4:**
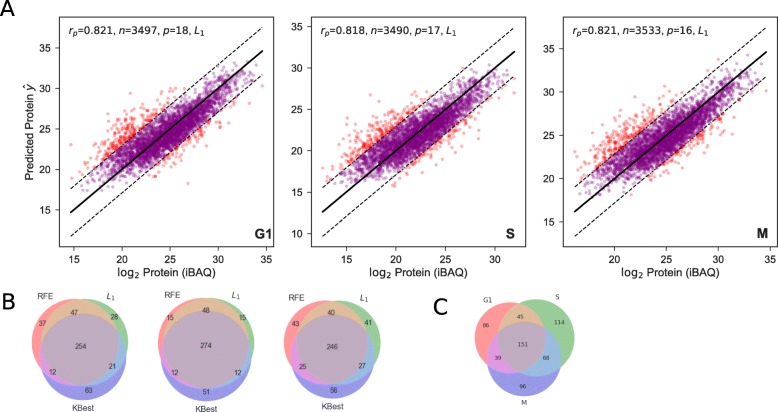
Biological interpretability in outliers between actual and predicted protein abundances. **a** Scatterplots of measured protein abundance (*y*) against *L*_1_-regularized predicted ($\hat y$), using gradient-boosted regression trees (GBRT) with LOOCV for G1, S and G2/M cell cycle. 90th percentile outliers with respect to the squared-error (*ε*) highlighted in red. *n*_*o*_ refers to the number of outliers. **b-c** Venn diagrams of outlier (red) overlap between RFE, *L*_1_ and K-Best feature selectors per cell cycle phase (**b**), and between G1, S and G2/M cell cycle phases per feature selector (**c**)

Using feature selection, we incorporated reduced input from *L*_1_ regularizer sets into GBRT models for G1, S and G2/M cell cycle phases, using Leave-One-Out Cross Validation (LOOCV) for each predicted gene (Fig. 4a), with significantly stronger Pearson product correlations (*r*_*p*_=0.82, *R*^2^=0.67) across all cell cycle phases than a naive predictor with just mRNA and translation inputs, therefore explaining two-thirds of protein variation. Vogel et al. [2] found similar findings, with features that focused on individual amino-acid frequencies, additional experimental data (such as mRNA decay rate) and codon-related features. They too found polyadenylation, GC content and codon bias index to be insignificant features, with strong negative correlations in coding sequence and 3’-UTR sequence length (refer back to Fig. 2). Previous work has demonstrated that short mRNAs tend to be more stable than long mRNAs [26] and are more efficiently translated; with the addition that resulting short amino-acid chains may fold into their tertiary structure faster than their longer counterparts. Other arguments stem from decreased translation initiation in long sequences [27], due to an increase in mRNA secondary structures found in longer 5’-UTR regions.

With perfect prediction lying on the *y*=*x* line (black), outliers signify difficult-to-predict proteins that according to our hypothesis are involved in post-translational modifications/processes, which we characterise using different percentiles with respect to the squared-error (*ε*, red). Indeed across all phases and feature selectors, we notice at least a 2:1 ratio of outliers lying above the regression line to below, indicating that the global model trained on all proteins tends to overestimate the abundance of some proteins when in fact they should be lower. This ratio is lower than Gunawardana [13] where the ratio was 23:1 above/below conducted using steady-state yeast models, therefore for this pattern to follow in a dynamic experiment is supportive of using novelty detection as a powerful theoretical principle. This would strongly suggest that post-translational modifications or degradation is taking place in these proteins which are not accounted for in our model input parameters. For proteins underestimated in abundance, this may be due to lack of resolution in only having three timesteps (six hours apart), detecting proteins without steady-state abundance, or time-lag concentration effects. Outlier overlap between feature selectors is reasonable (see Fig. 4b), with roughly two-thirds of proteins identified as 90th percentile outliers across RFE, *L*_1_ and K-Best feature selectors, to improve robust identification of outlier proteins. In addition to this, there is surprising overlap between cell cycle phases (Fig. 4c), with roughly one-quarter of proteins found to act as outliers across all 3 phases, with roughly double S-G2/M outliers compared to G1-S or G2/M-G1 outliers, across multiple percentiles.

Across 90th percentile outlier proteins, ZNF687 and CTNNB1 (both above prediction line) occur in the top 5 outliers with highest *ε* across all 3 phases, with many proteins not fluctuating much in terms of *ε* across the cell cycle. In addition to choosing 90th percentile outliers, we examined the outlier overlap of 95th, 97.5th and 99th percentiles which demonstrate a similar pattern to Fig. 4c, although there is a gradual drop in proportion of shared proteins in all 3 phases due to the decrease in sample size (see Additional file 4). We contrasted this to 5th percentile proteins (most accurately predicted), where there is very little overlap in outlier proteins across all 3 cell cycle phases (one in 100), as one would expect if proteins were randomly sampled.

### Evidence of post-translational modification/degradation in outliers reveals new insights

**Fig. 5:**
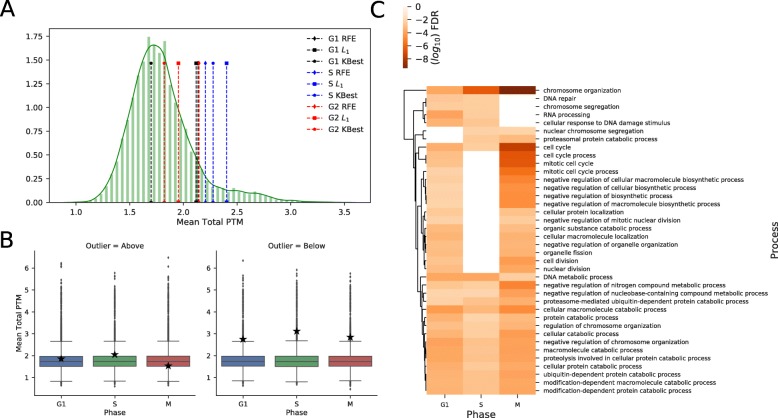
Coarse and Fine PTM analysis reveals protein modification/degradation as outliers. **a** Histogram of 10000 bootstrap subsamples of mean total post-translational modification (PTM) prediction sites versus sample outlier sets (lines), using 90th percentile. **b** Boxplot distributions of 10000 bootstrap subsamples of total mean PTM versus 90th percentile outliers (black star) above and below predictor line. **c** Hierarchical clustering of selected Gene Ontology Biological Process (GOBP) terms using (*log*_10_) *p*-value FDR with Benjamini correction (*p*<0.01), in 90th percentile

To contrast our hypothesis of post-translational modification (PTM) in outlier proteins, we generated structural site predictions of Acetylation, Methylation, Palmitoylation, Phosphorylation and Sumoylation for each amino-acid sequence. We then calculated the total number of PTMs for each protein and compared the outlier mean total PTM to 10000 mean total PTMs from randomly sub-sampled protein sets of the same size (Fig. 5a). In all upper 90th percentile sets we examined, we found the vast majority of outlier sets to have a mean PTM score greater than the mean of the distribution, with S phase consistently lying furthest from the mean (see Additional file 5); thus indicating that outliers found in our regressors are more likely to have significantly more post-translational modification sites. Despite this, paired *t*-tests between outlier and random-sampled sets revealed that only around 15-20% of tests yielded a *p*-value <0.05, meaning we could not reject the null hypthesis. To explore differences between outliers above and below the prediction line, we split the dataset as we would expect these groups to differ in functionality. Interestingly, proteins below the regression line consistently have a higher mean total PTM value than outlier proteins above the regression line, with some passing the 95th-confidence threshold (Fig. 5b). These are proteins that are underestimated by our predictor, thus these modifications likely play a role in protein stability and post-transcriptional regulation, and indeed acetylation is known to stabilise proteins post-translation. It is interesting that significant PTMs are not seen in outliers that overestimate protein levels; this would suggest that most PTMs are not marking their respective protein for degradation but modifying the protein role in it’s interaction to the external environment. Given that the most frequent PTM site found is Phosphorylation, which is known to have a vast array of roles, causing degradation [28] in some proteins, and activation/promotion [29, 30] such as p53 phosphorylation in others; this makes inferring function from PTM sites alone difficult.

To consider deeper functional roles than just exploring the counts of PTM sites (considered our ’coarse level analysis’), we perform Gene Ontology Biological Process (GOBP) enrichment analysis on 90th percentile outliers for each cell cycle phase (see Additional file 9) and clustered them in terms of their term significance/occurrence (Fig. 5c). We filtered for GOBP terms that had an FDR value <0.01 across at least 2 cell stages. G2/M phase contained the largest number of significant terms identified, with strong evidence for post-translation degradation pathways found in protein catabolic process/ubiquitin-dependent catabolic process terms (bottom of cluster), across all 3 cell cycle stages. Alongside this, we also found strong significance in (negative regulation of) chromosome organization across all phases, suggesting a strong relationship between chromatin modelling and post-translational modifications/degradation with associated proteins. Indeed, we found strong presense of helicases (HEL-), ATAD2 and E2F4/5 in all outlier sets, known to have roles in DNA repair/chromatin-modifying proteins [31]. Further to this, the presense of many (regulation of) cell-cycle related terms between G2/M-to-G1 stages indicates that post-translational modification/degradation contributes significantly in robust control of cell cycle factors; perhaps more than previously expected. The gene regulation network within the yeast cell cycle have already been explored in detail [32], and highlights the fact that although over 800 yeast genes are involved in the process, a significantly smaller portion are responsible for regulating the cell cycle.

We performed further enrichment analysis on outliers found above and below the regression line, wherein with above outliers; protein catabolic/proteolysis terms to exist only in M-G1 stages, with cell cycle/division/chromosome segregation across all 3 stages, with DNA repair/response to DNA damage found shared between G1-S. Contrasted to below outliers; we found dominance of post-transcriptional regulation terms and translational frameshifting across all 3 stages, with RNA/mRNA stability found in S-G2/M groups, and RNA processing/regulation of RNA splicing found in G1-S (see Additional file 6).

Whilst there is strong support for post-translational regulation independent of time, there may be bias from time-lagged mRNA/translation expression that was transcribed at a previous timestep unaccounting for the change in subsequent protein expression, as explored by other authors [33] using systems biology simulations. To account for this we developed predictive models which incorporated mRNA/translation expression at a previous timestep in the cell cycle rather than at current time. Changes in correlation between normal (mRNA and translation at time *t*) and lagged (*t*−1) expression are minimal (see Additional file 7). Further to this, roughly 90% of outlier proteins overlap across all three phases between mRNA at time *t* and time *t*−1, demonstrating a small but insignificant change in predictions generated from mRNA time-lag.

## Discussion

### Analysis of time-series concentration with sequence-derived features

In this work we have collated time-series concentrations of mRNA, translation and protein from Aviner [8] and sequence-derived features from other sources [23, 38]. Consistent with previous authors, our data shows that mRNA and translation go some way in explaining protein variation (*R*^2^=0.23 and 0.45). This diverges from previous similar work by Schwanhäusser et al. [35], where protein translation is calculated using a mathematical model of mRNA and protein rates, rather than measured directly; and where sequence derived features are not factored in their analysis. Our data establishes the redundancy of using mRNA level as a proxy to protein level with the introduction of translation measurements via PUNCH-P [9], likely due to factoring in post-transcriptional controls as translation occurs after mRNA processing. The remaining discordance in correlation between translation and protein is therefore mostly associated with post-translational regulation of protein abundance once synthesised.

To improve predictive power, we extracted features about physical properties associated with the underlying mRNA/amino acid sequence such as CAI, tAI and gene length. Clustered inter-correlation analysis between features showed groupings of features usually by function (i.e strong correlation between mRNA and amino-acid length). Negative correlations between sequence length and protein level have been similarly reported in studies of other organisms [2], and is theoretically supported. However codon bias correlations (CAI, tAI) to protein are noticeably smaller than in previous studies [13, 43], which may be due to further robustness of the gene regulatory framework in *H. sapiens* compared to *S. cerevisiae*, or due to recording dynamic time-series nature of the data rather than a steady snapshot.

To simplify the model (and prevent overfitting), we considered unsupervised learning techniques, particularly PCA and t-SNE which underperformed, due to the complex interactions occurring between the features. Whilst other applications for dimensionality reduction often have significantly higher dimensions p, such as image or natural language processing; we found many features contributing a small but significantly cumulative reduction in model error. This highlights the diverse low-impact optimizations that exist in the cellular framework for self-modulation, whether by sequence length, codon bias, translational efficiency or other pre-translational methods in each associated mRNA.

### Predicted outliers indicate post-translational regulation

Supervised learning on the input features enabled a linear comparison between actual and predicted protein concentrations, where we inferred that proteins furthest from the linear model are involved in biological processes which are primarily regulated post-translation. Choosing the most appropriate percentile to identify outliers is not clear; Gunawardana et al. [13] chose a 2.5% cutoff, but had a small number of outliers (<=50). We chose a 10% (90th) cutoff in order to improve the significance of subsequent GO analyses, at the cost of possibly including proteins that may not be deemed as outliers. Modest overlap (25-40%) between outlier proteins across the cell cycle shows a core group of proteins that the model fails to predict consistently, which is enriched for catabolic processes.

In relation to effects from time-delayed mRNA expression, we found that it partially affects 10-12% of proteins we’ve sampled by bootstrapping, but due to low time-resolution with only three steps in the cell cycle, this conclusion is drawn with caution as a 6-h time delay window is more than sufficient for mRNA expression levels to change aberrantly.

## Conclusions

This work has expanded on previous multi-’omic expression data and integrated the concept of novelty detection by outliers to provide insights into post-translational modification and degradation through data-driven modelling of the human cell cycle, with potential applications in more completely predicting protein abundance at certain timesteps in normalcy. This lends to a powerful preprocessed dataset being made publicly available forming a benchmark for predictive proteomic studies. Of particular interest is the separation between extensive protein modification found to be underestimated, and protein degradation overestimated by our model. We have explored the practicalities with selecting powerful features in protein prediction, and we have reduced the space over which experimental exploration is needed and provided evidence of biological functionality to be confirmed experimentally.

## Materials and methods

### Data retrieval

Human HeLa cell cycle data was taken from Aviner et al. [8], with triplicative measurements for mRNA, translation and protein, for which the empirical mean is taken. mRNA data is pre-normalized using robust multi-array average (RMA) [34], whereas translation and protein are pre-normalized using intensity-based absolute quantification (iBAQ) [35]. These experiments were normalized at the experimental level by analysing the same amounts of biological material at each cell cycle phase. Messenger-RNA transcript variants and related meta-information were extracted from NCBI Entrez Direct [23, 36] via Biopython v1.7 [37] package in Python 3.6. Unique Gene names (HGNC) [24] from the cell cycle dataset were mapped to NCBI Accession Numbers from RefSeq curated dataset (beginning with NM_), obtaining GenBank files [38] for all mRNA transcripts associated with HGNC gene names. Exon data and elements from the feature table were extracted and counted. In addition to this, we retrieved the associated curated protein transcripts (NP_) to each translated mRNA product found in the coding-sequence section of the feature table.

### Feature extraction

The coding sequence (CDS) is derived using mRNA sequence and exon range information, we filter out transcripts where the calculated coding sequence (in terms of mRNA) when translated does not match the amino-acid sequence found in the GenBank file. We count the number of exons, sequence-tagged sites (STS), miscallaenous features, regulatory regions and poly-adenylated tails in the mRNA transcript feature table; in addition to the number of protein sites, regions and predicted molecular weight (PMw), per protein product (NP_) linked to transcript files. We used Biopython [37] to derive mRNA GC content and handle DNA/amino acid sequences. We extracted CAI and ’the effective number of codons’ (Nc) using CAIcal [39] server (http://genomes.urv.es/CAIcal/), using CDS sequence as input in conjunction with the Human Codon Usage table as frequencies per thousand (http://genomes.urv.es/CAIcal/CU_human_nature) from the Ensembl database (Release 57). We used ExPASy’s ProtParam [40] module in Biopython to predict pI, Aromaticity, Instability Index, GRAVY and protein secondary structure features (helix, sheet, coil). tAI values are calculated using stAIcalc [41], using the offline version with human tRNA gene copy numbers taken from GtRNAdb [42] for hg19 (NCBI build 37.1 Feb 2009). CUB (relative codon usage) is calculated following the method in [43], which does not require a reference codon usage table. The change in Gibbs Free folding energy *ΔG* in the 5’-untranslated region, indicating the amount of mRNA secondary structure features, is predicted using the offline RNAstructure EnsembleEnergy algorithm [44]. Predictions for post-translational modification sites for phosphorylation, methylation, sumoylation, palmitoylation and acetylation are made using the PTMs Peptide Scanner (PPS 1.0) [45], using the batched offline tool with the amino-acid sequence as input.

### Preprocessing

Due to a protein being encoded possibly by more than one mRNA transcript (transcript variants), to effectively map mRNA sequence-derived features to the cell cycle, we select the longest mRNA transcript for each protein, and merge this into the cell cycle dataset leading to a dataset of 6592 proteins; with roughly 3500 proteins containing no missing values. We scaled the count features such as the number of exons by the mRNA sequence length (or equivalent for amino-acid count data) to obtain a relative frequency mitigating sequence-length bias. PTMs from PPS 1.0 are grouped by the type of PTM per protein and integrated into the cell cycle set by NCBI accession protein number (NP_), with missing values assumed to be zero (filled).

### Feature and model selection

All of the machine learning algorithms/feature selectors are encapsulated in Scikit-Learn [46] within Python. Feature selection is an important preprocessing step in removing redundant features that could negatively impact the coefficients of any downstream model produced, and to reduce the dimensionality of the problem. We used RFE [47], *L*_1_-induced regularization [48] and SelectKBest/ANOVA as three separate methods, but we will only cover *L*_1_ here as it is the primary selector for all of the figures in this paper. LASSO is an extension to ordinary least squares (OLS) in that it applies an *L*_1_-norm penalty to the objective minimization function [48], as shown here in matrix notation:

$$\mathbf{\min}\left\{\|Xw-y\|^{2}+\alpha\|w\|_{1}\right\} $$

where $\mathbf {X} \in \mathbb {R}^{n,p}$ refers to the input matrix (with bias term), $\mathbf {y} \in \mathbb {R}^{n}$ refers to the target vector, with $\mathbf {w} \in \mathbb {R}^{p}$ as weights of unknowns. ***α*** controls the level of regularization and *L*_1_-norm tends to produce sparse solutions of ***w*** when *α* is large. The selection of *α* is described in Fig. 3 and is mostly a hyperparameter to be tuned according to the level of sparsity you wish to induce. Gradient-boosting (GBRT) is a non-linear tree-based method for combining many weaker decision tree learners into a single strong learner and is described in detail here [49]. We use a large number of base estimators (1000) for all GBRT models, with a relatively small learning rate (0.01) which in general trades off computational power for higher accuracy. GBRTs are also known to be fairly robust to overfitting, and for protein prediction we use leave-one-out cross validation (LOOCV) for deterministic out-of-sample testing. We selected outliers with respect to our model by looking at the squared-residuals:

$$\epsilon_{i}=(y_{i}-\hat y_{i})^{2} $$

where *y*_*i*_ represents our actual protein level and $\hat y_{i}$ is our predicted level. We explored the 5th, 90th, 95th, 97.5th and 99th percentiles within *ε*.

### Bioinformatic analysis

Statistical analysis of the pairwise monotonic relationship (*r*_*s*_) between features uses Scipy 1.0 [50] and we use Spearman-rank correlation between features as we do not assume a linear relationship. For comparisons between measured and predicted protein abundance, we use Pearson’s product moment correlation (*r*_*p*_) as we assume a linear relationship between variables that are (meant to be) the same. We use ($R_{p}^{2}$) when we wish to compare to other studies that have also used *R*^2^ to describe model accuracy. Measurements for mRNA, translation and protein are presented as means across triplicative measurements, with ±*SD* where indicated. Paired t-tests used in checking for significance in PTM outlier samples was conducted using Scipy. Hierarchical clustering was done automatically to matrix inputs using Seaborn 0.8.1, using clustermap. For Gene Ontology Enrichment analysis, we used the ToppGene suite [51], using FDR <0.01. For clustermaps of GO analysis, we filtered for terms that were found in 2 or more cell cycle phases.

## Supplementary information

**Additional file 1** mRNA comparison between genes with and without missing translation measurements. Points with (blue circle, *r*_*s*_=0.23-0.24) and without (red triangle, *r*_*s*_=0.46-0.48) missing translation data. Linear model (black) with mean centre of cluster (shape refers to group).

**Additional file 2** Naive linear predictor of protein using mRNA and translation. Scatterplots of measured (*y*) versus predicted ($\hat y$) protein across G1, S and G2/M cell cycle phases, with Spearman-rank correlation *r*_*s*_, sample size *n* and number of parameters *p*.

**Additional file 3** Selecting algorithm with highest correlation using GridSearch 10-fold cross validation. Barplot representation of different algorithms for training score (right) and testing score (left). Gradient-boosted regression trees (GBRT) performed best across all phases. ±SD indicate cross-validation scores.

**Additional file 4** Outlier overlap for all feature selectors across q5, q90, q95, q97.5 and q99. Venn diagrams across RFE (left), *L*_1_ (middle) and KBest (right) feature selectors, with vertical representing n-th percentiles q5, q90, q95, q97.5, q99 respectively (venn-phase-95.png).

**Additional file 5** Distributions of random-subsampled PTM sites versus. outlier PTM sites. Histogram of 10000 bootstrap subsamples of mean total post-translational modification (PTM) prediction sites versus sample outlier sets (vertical lines), using 90th, 95th, 97.5th and 99th percentiles.

**Additional file 6** Hierarchical Clustering of GOBP Terms above (left) and below (right) the regression line (see Fig. 4c). using (*log*_10_) *p*-value FDR with Benjamini correction (*p*<0.01). Annotated circles (orange) pseudo-group regions of interest for each plot. Dendrograms aside each plot identify grouped-distance.

**Additional file 7** Scatterplots of protein levels against predicted protein $\hat p$ generated from different mRNA/translation measurement inputs. a) *mRNA*_*t*_ b) *mRNA*_*t*−1_ c) *translation*_*t*−1_ or d) *mRNA*_*t*−1_,*translation*_*t*−1_. From top: S, G2/M, G1 cell cycle phase. Yellow plots refer to the normal model (see Fig. 4a). Cell cycle terms are annotated for using Gene Ontology and overlaid with correlation. *t* refers to the cell cycle step (G1, S or G2/M).

**Additional file 8** Table of expanded feature names with abbrievations. Includes input feature abbrievations used in Fig. 2.

**Additional file 9** Complete GO Analysis for 90th percentile outliers. We only make use of Biological Process and FDR < 0.01, but many other categories are included with this analysis. Tabs include All 9 (3 selection features * 3 cell phases), G1, S and G2/M.

**Additional file 10** Combined dataset. Dataset of Aviner’s work (log2 mRNA, translation and protein abundance for G1-S-G2/M) with gene, mRNA and amino-acid sequence derived features and associated labels.

**Additional file 11** Feature Selection procedure. Description of the feature selection process for RFE, *L*_1_ and Select *k*-Best, including parameter choices in this pdf.

## Data Availability

All processed files are submitted as supplementary material. Cell cycle expression data can originally be obtained from Aviner et al. [8]. Other sources (such as sequence-derived features) can be originally obtained from their respective open-access databases. See the Materials and methods section for further details.

## Abbreviations

CAI: Codon adaptation index
CDS: Coding sequence
CV: Cross-validation
ER: Evolutionary rate
G1/S/G2/M: Refers to timesteps in the cell cycle, often simply referred to by their abbrievation
GBRT: Gradient-boosted regression tree
GOBP: Gene ontology biological process
HGNC: Human genome nomenclature committee
MS: Mass spectrometry
MSE: Mean-squared error
PCA: Principle component analysis
pI: Isoelectric point
PMw: Protein molecular weight
PTM(s): Post-translational modification
PUNCH-P: PUromycin-associated nascent CHain Proteomics
t-SNE: t-distributed stochastic neighbour embedding
tAI: tRNA Adaptation index
