# Supplementary figures and images for: Uncovering extensive post-translation regulation during human cell cycle progression by integrative multi-’omics analysis
Source: BMC Bioinformatics. 2019 Oct 29;20:536. doi: 10.1186/s12859-019-3150-5 (PMC6820968; doi:10.1186/s12859-019-3150-5)

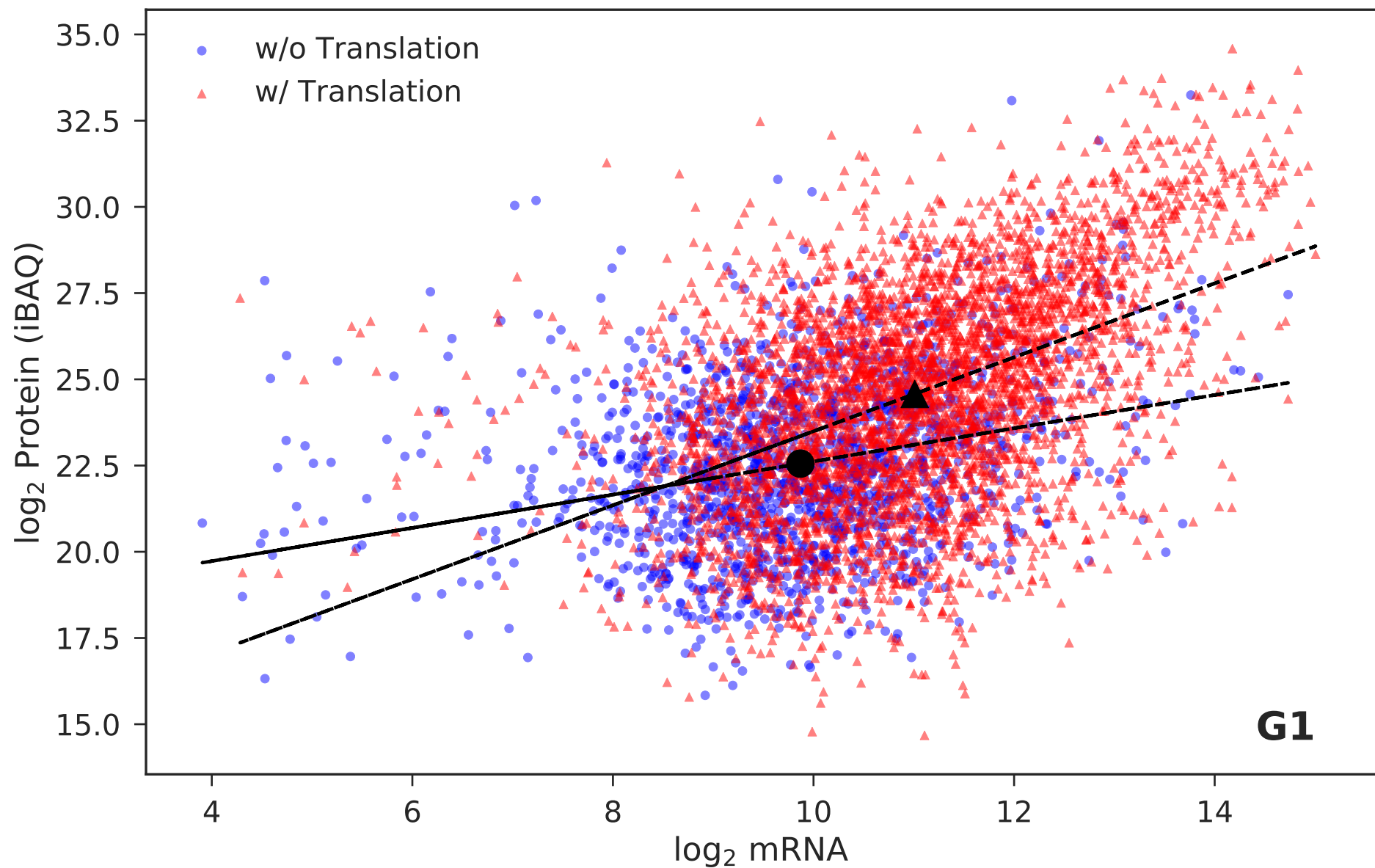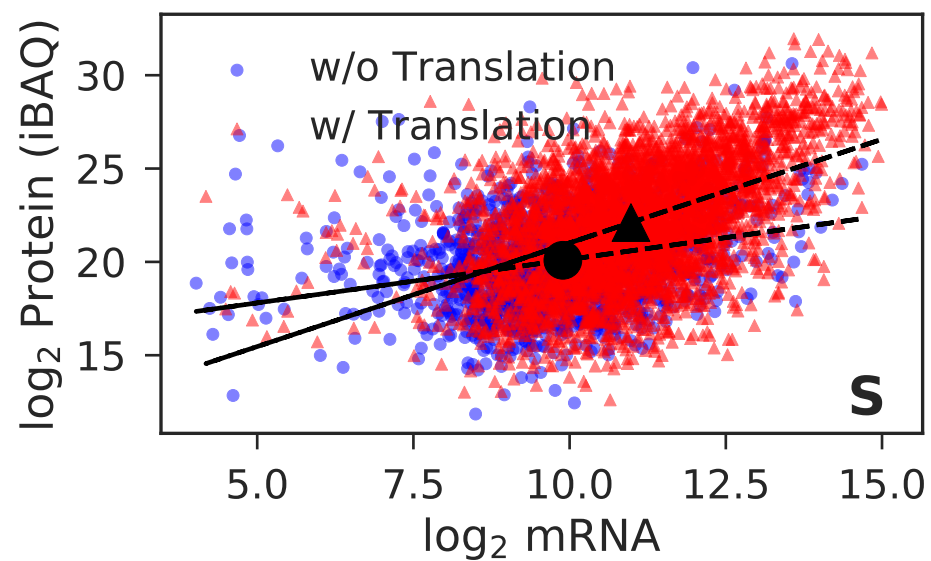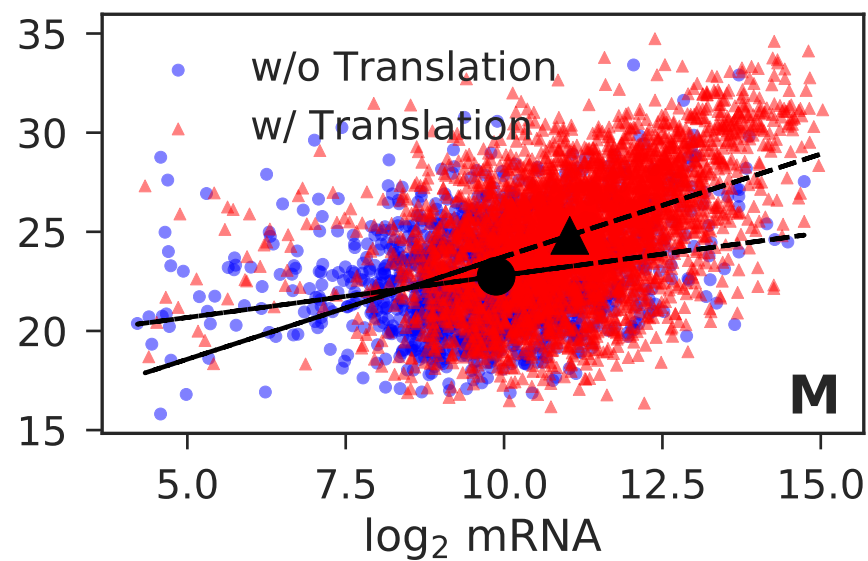

Supplement: Supplementary file 1 — Additional file 1 mRNA comparison between genes with and without missing translation measurements. Points with (blue circle, rs=0.23-0.24) and without (red triangle, rs=0.46-0.48) missing translation data. Linear model (black) with mean centre of cluster (shape refers to group). [file 12859_2019_3150_MOESM1_ESM.pdf]

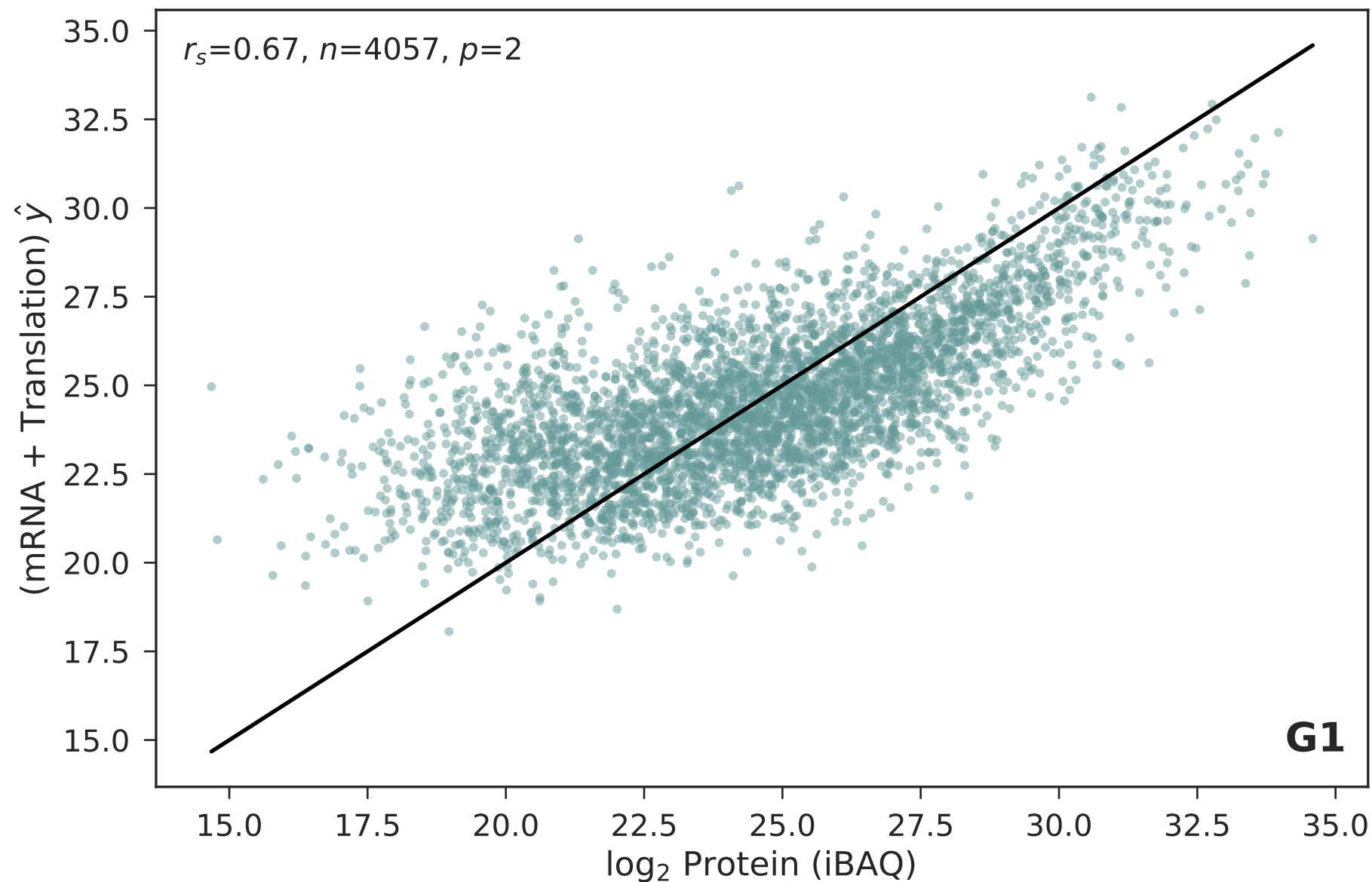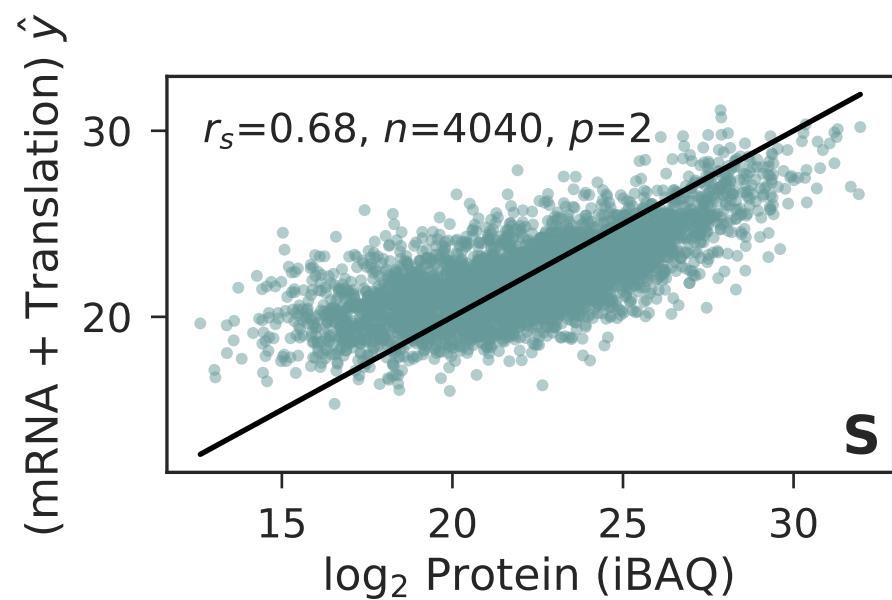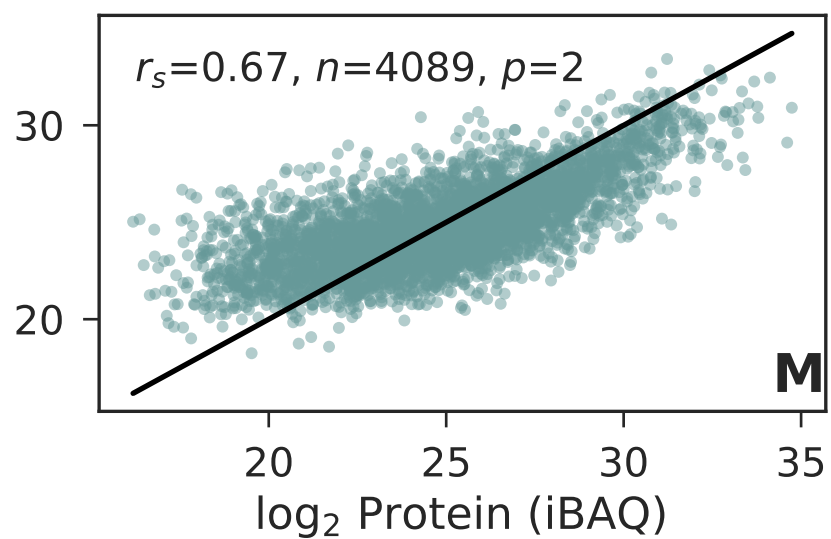

Supplement: Supplementary file 2 — Additional file 2 Naive linear predictor of protein using mRNA and translation. Scatterplots of measured (y) versus predicted ($\hat y$ŷ) protein across G1, S and G2/M cell cycle phases, with Spearman-rank correlation rs, sample size n and number of parameters p. [file 12859_2019_3150_MOESM2_ESM.pdf]

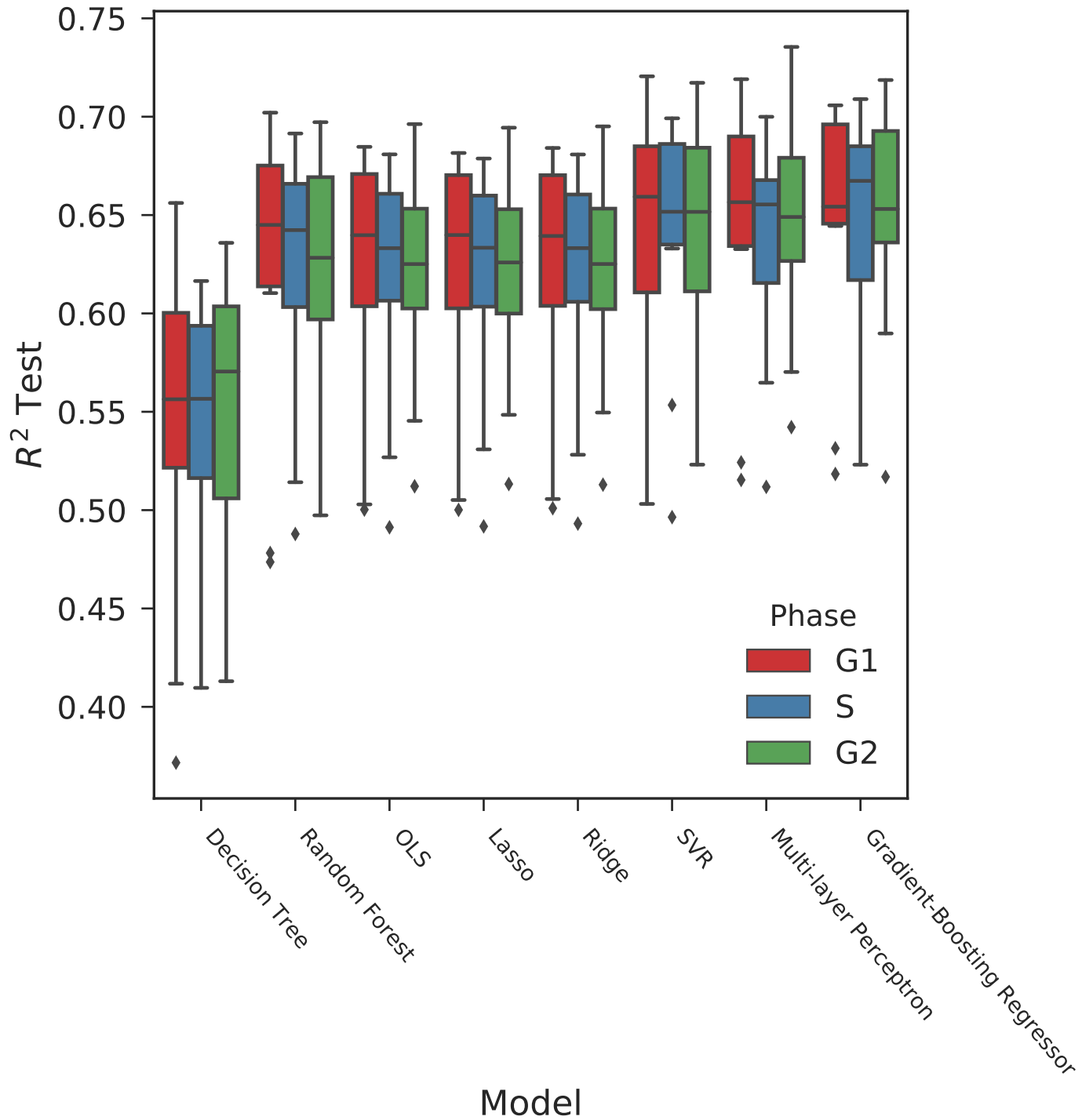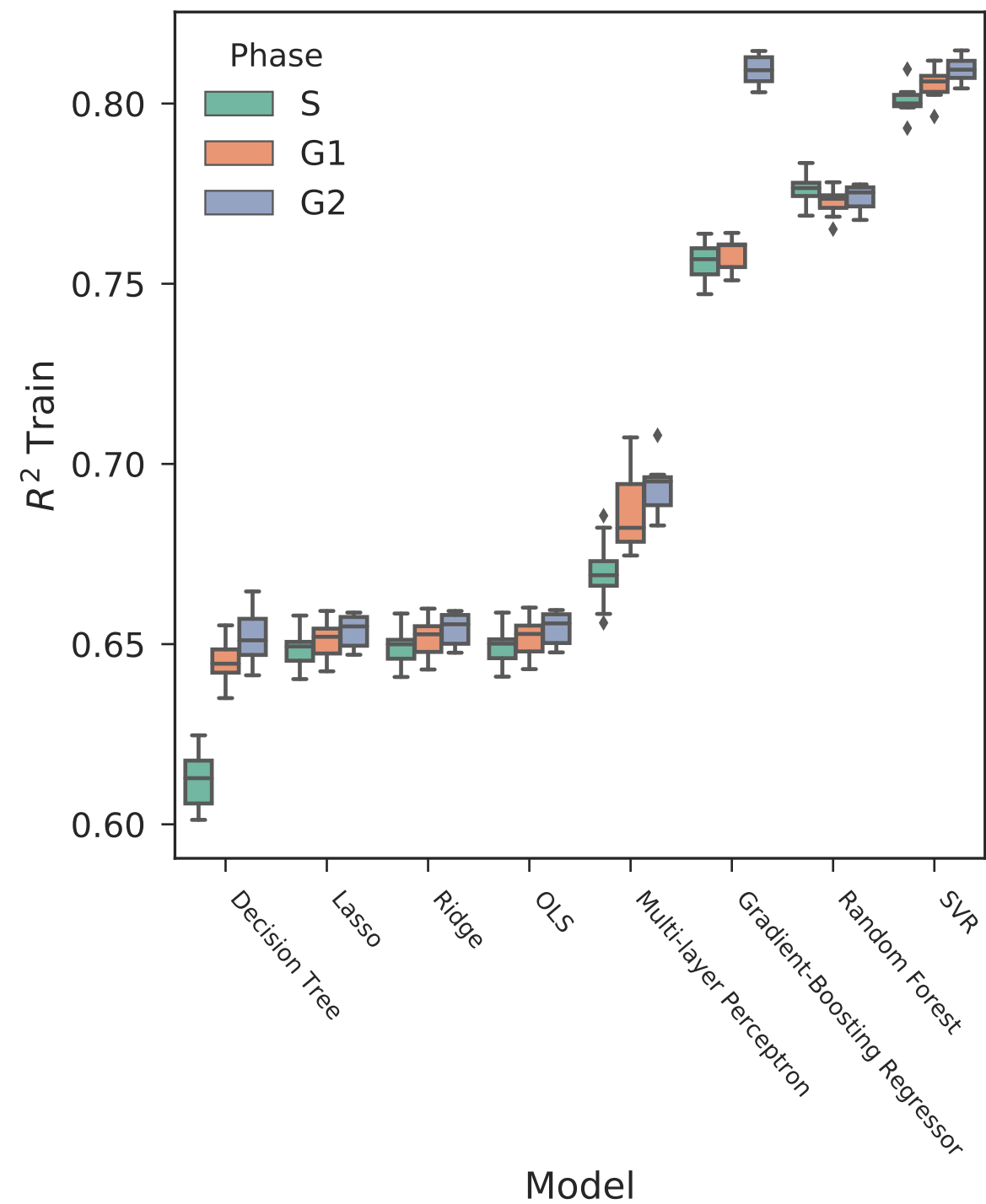

Supplement: Supplementary file 3 — Additional file 3 Selecting algorithm with highest correlation using GridSearch 10-fold cross validation. Barplot representation of different algorithms for training score (right) and testing score (left). Gradient-boosted regression trees (GBRT) performed best across all phases. ±SD indicate cross-validation scores. [file 12859_2019_3150_MOESM3_ESM.pdf]

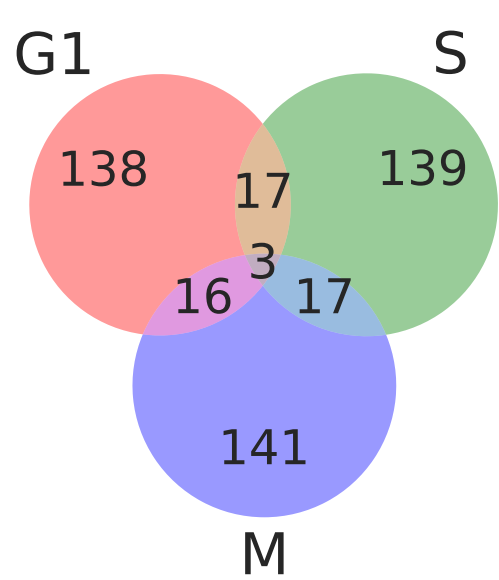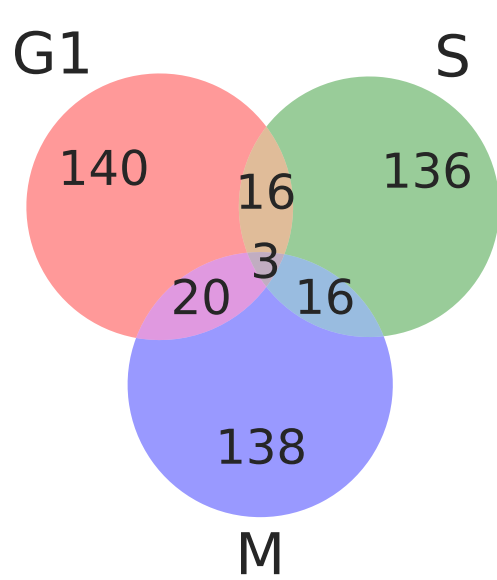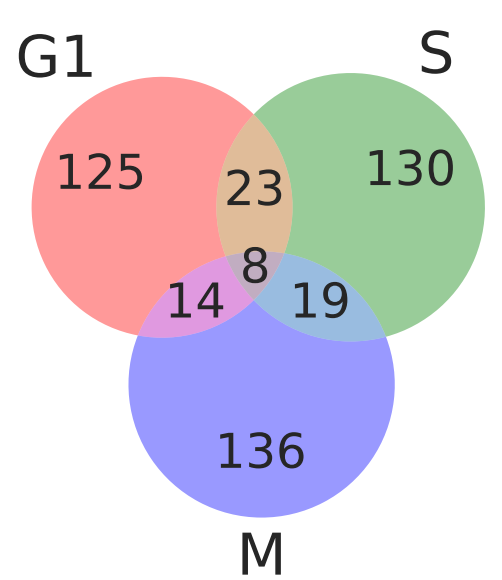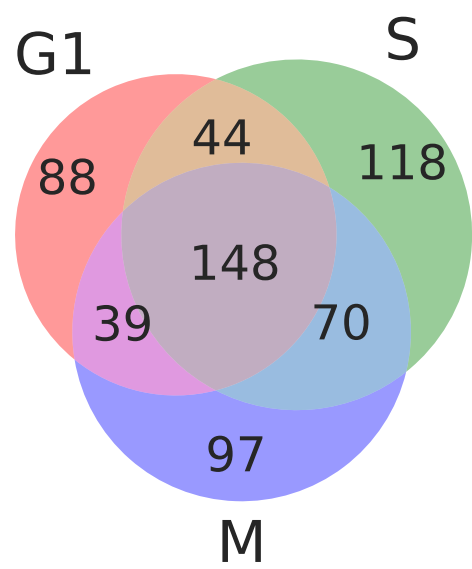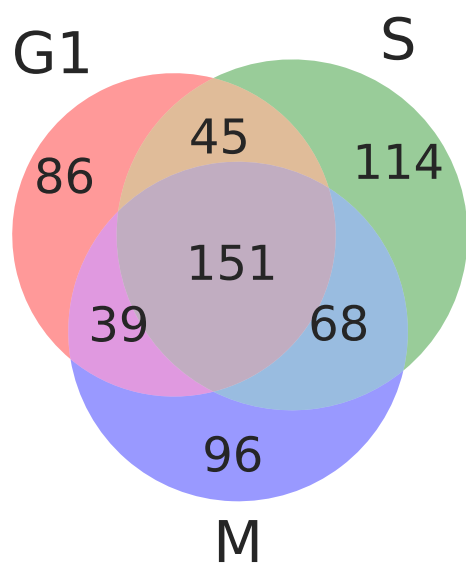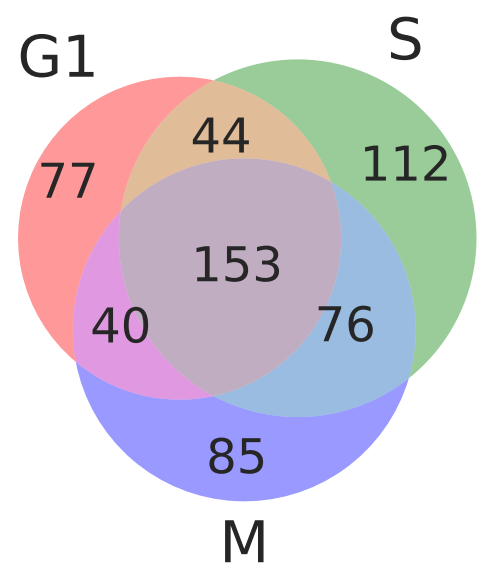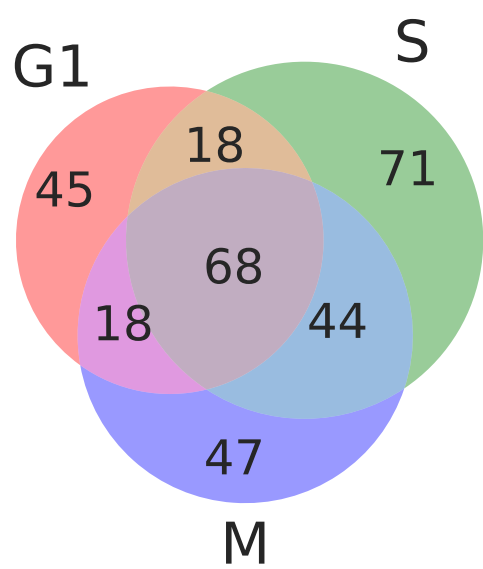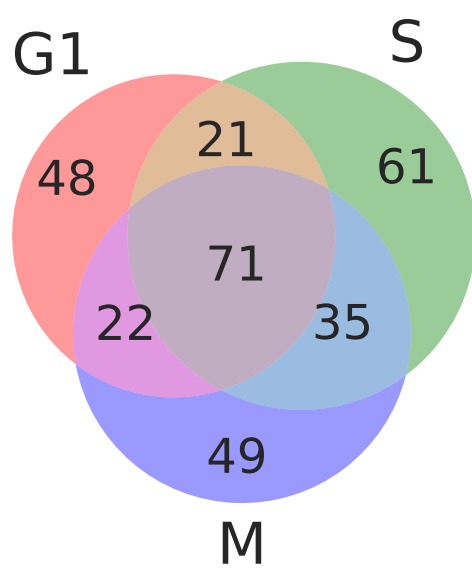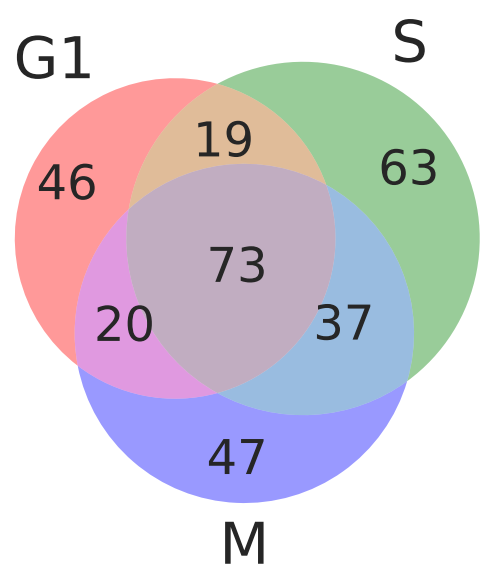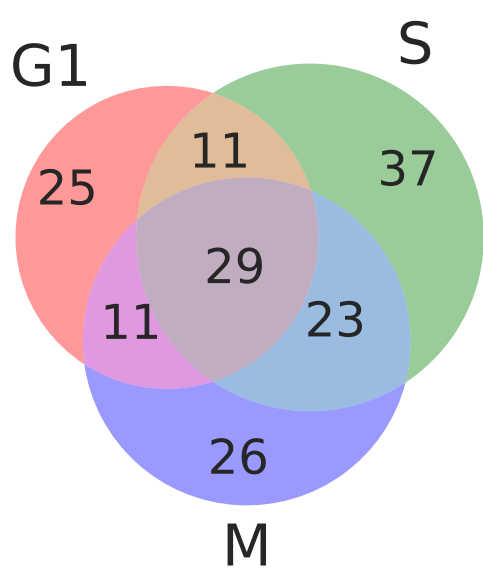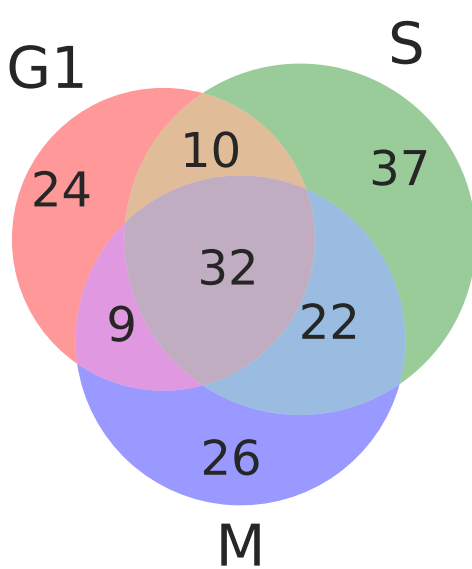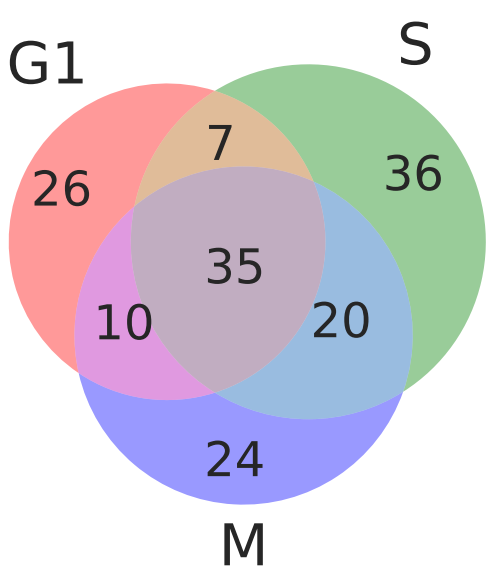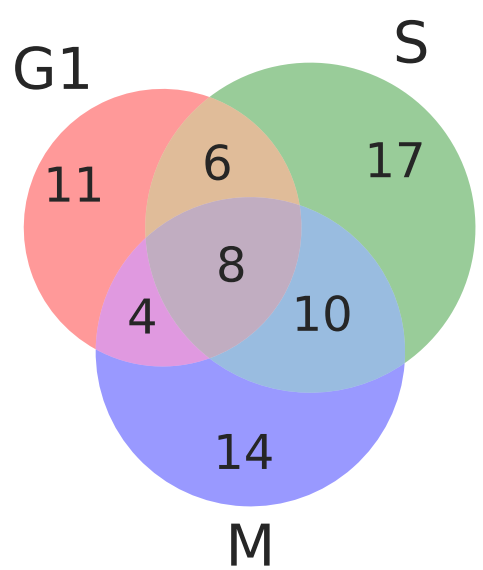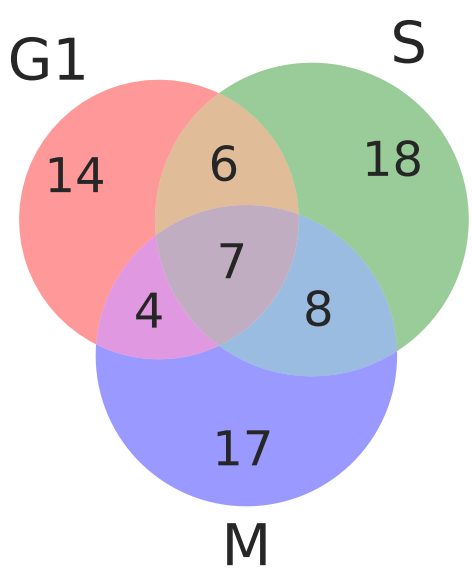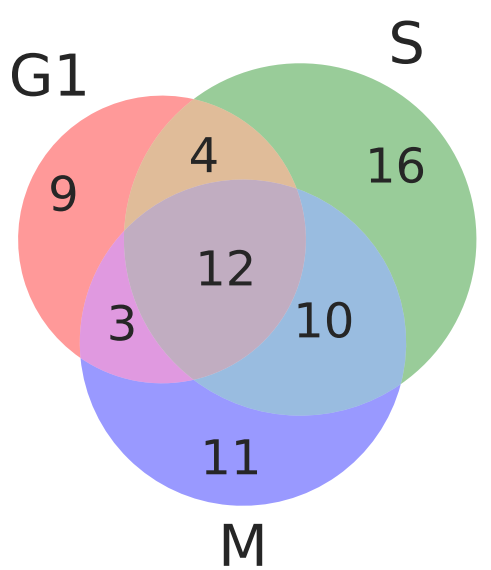

Supplement: Supplementary file 4 — Additional file 4 Outlier overlap for all feature selectors across q5, q90, q95, q97.5 and q99. Venn diagrams across RFE (left), L1 (middle) and KBest (right) feature selectors, with vertical representing n-th percentiles q5, q90, q95, q97.5, q99 respectively (venn-phase-95.png). [file 12859_2019_3150_MOESM4_ESM.pdf]

90th

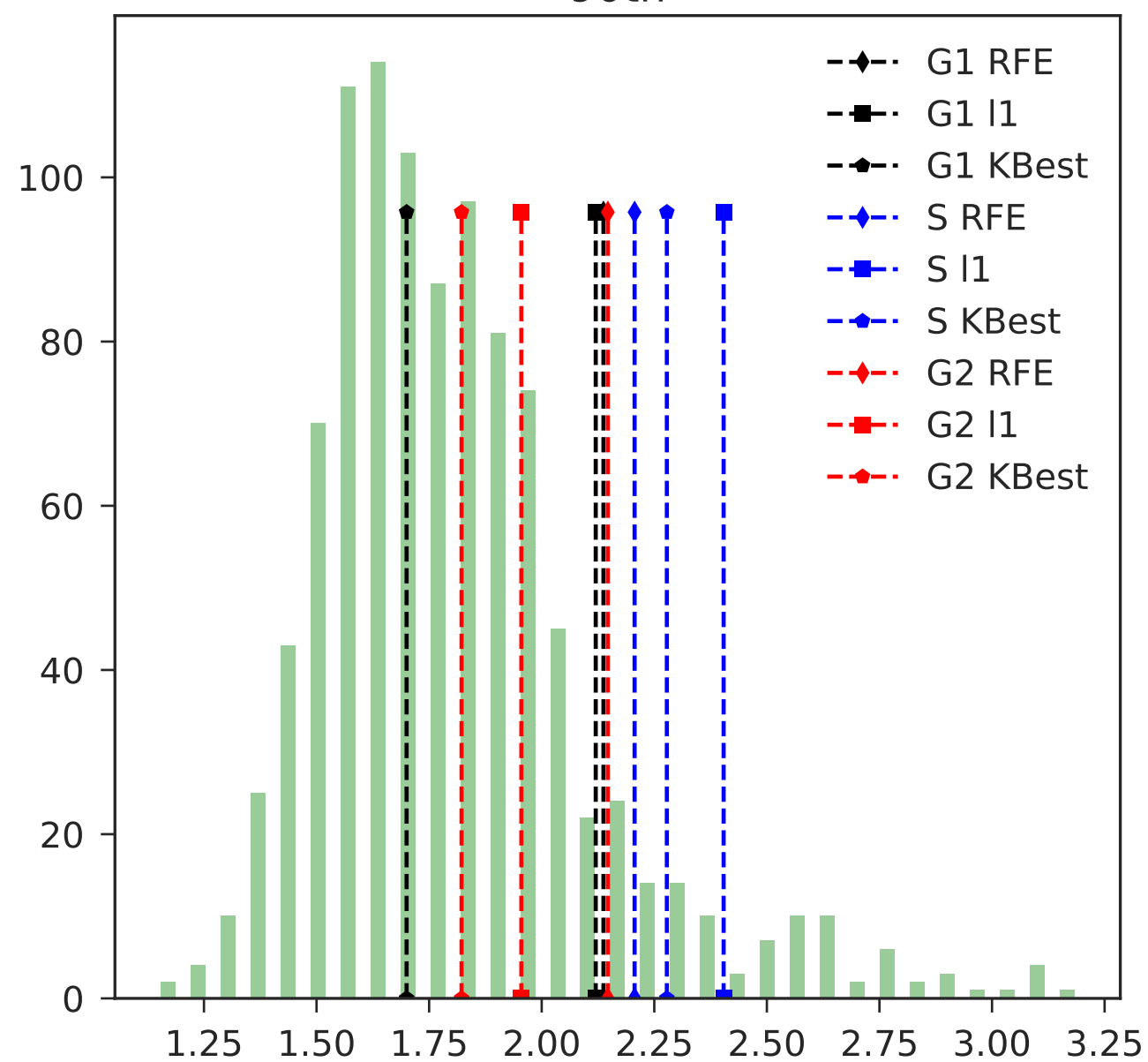

95th

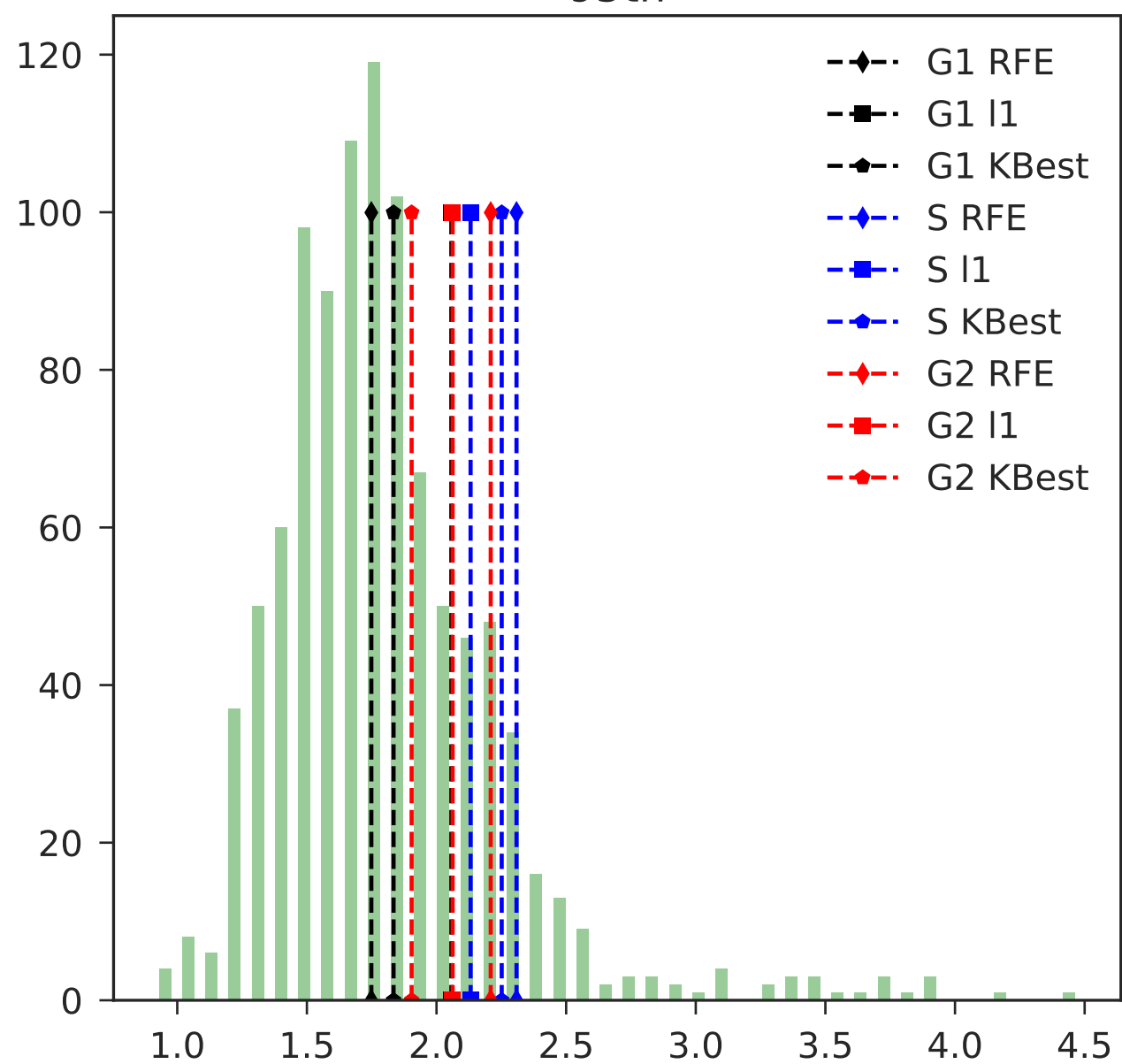

97th

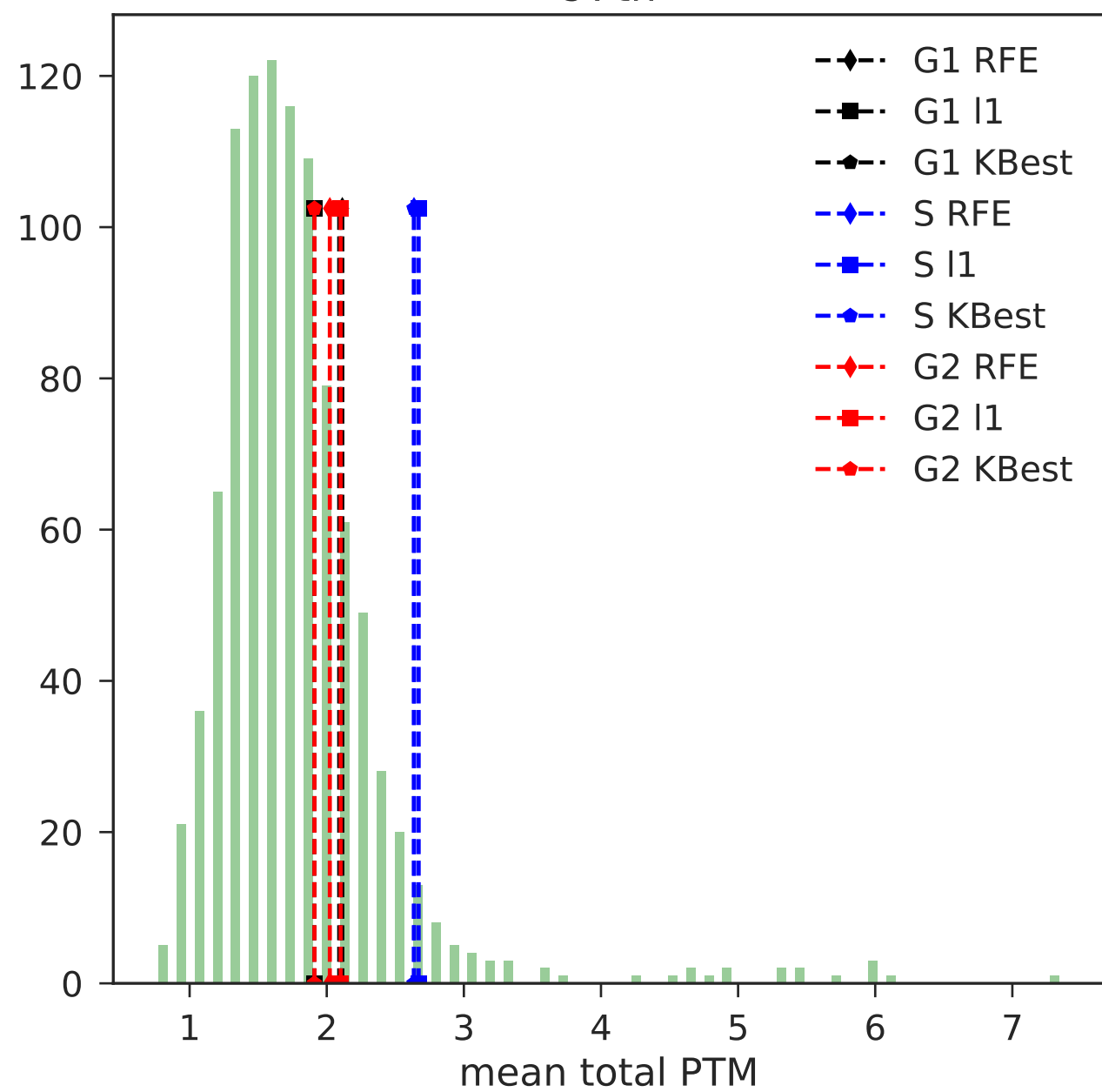

99th

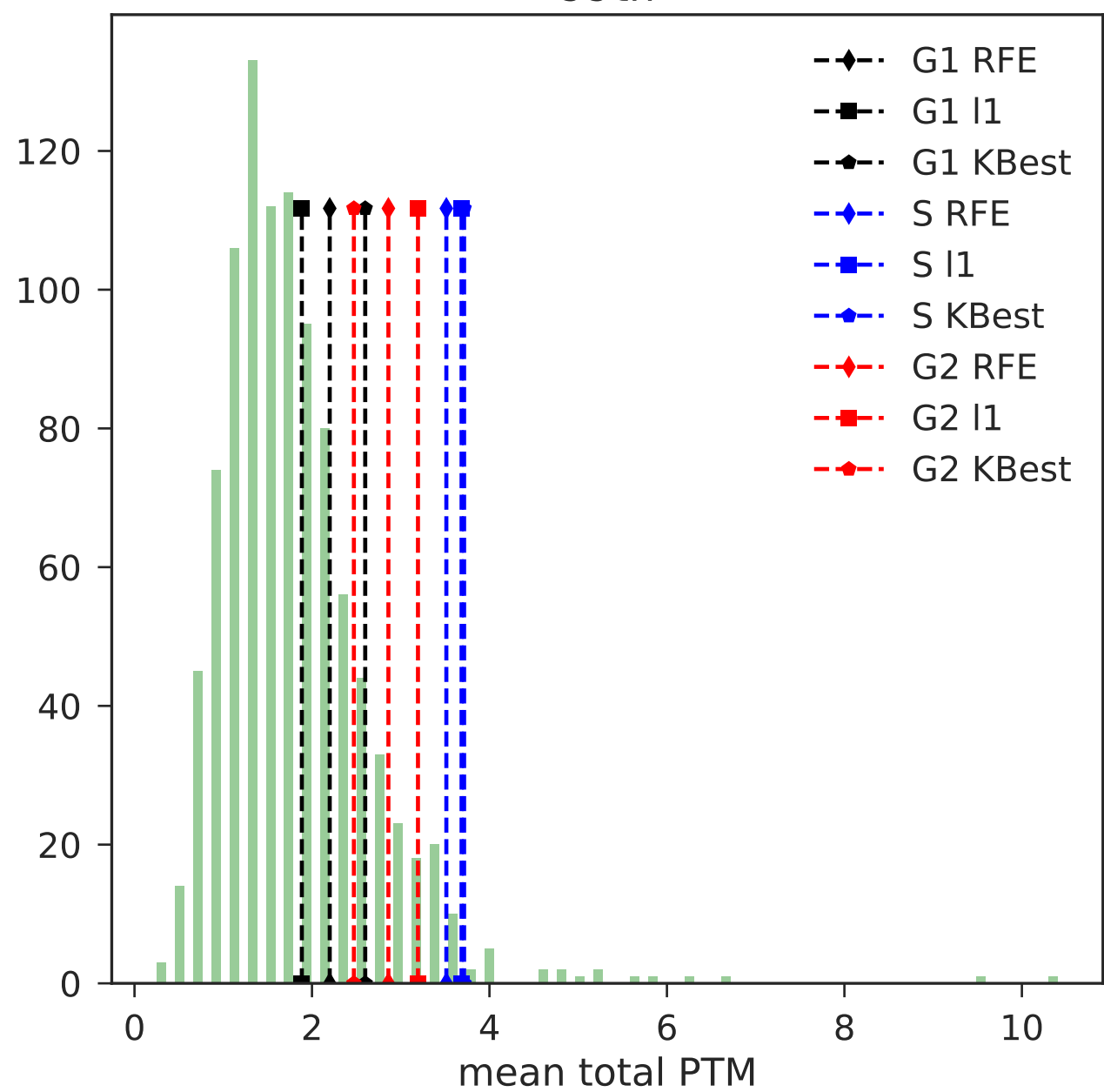

Supplement: Supplementary file 5 — Additional file 5 Distributions of random-subsampled PTM sites versus. outlier PTM sites. Histogram of 10000 bootstrap subsamples of mean total post-translational modification (PTM) prediction sites versus sample outlier sets (vertical lines), using 90th, 95th, 97.5th and 99th percentiles. [file 12859_2019_3150_MOESM5_ESM.pdf]

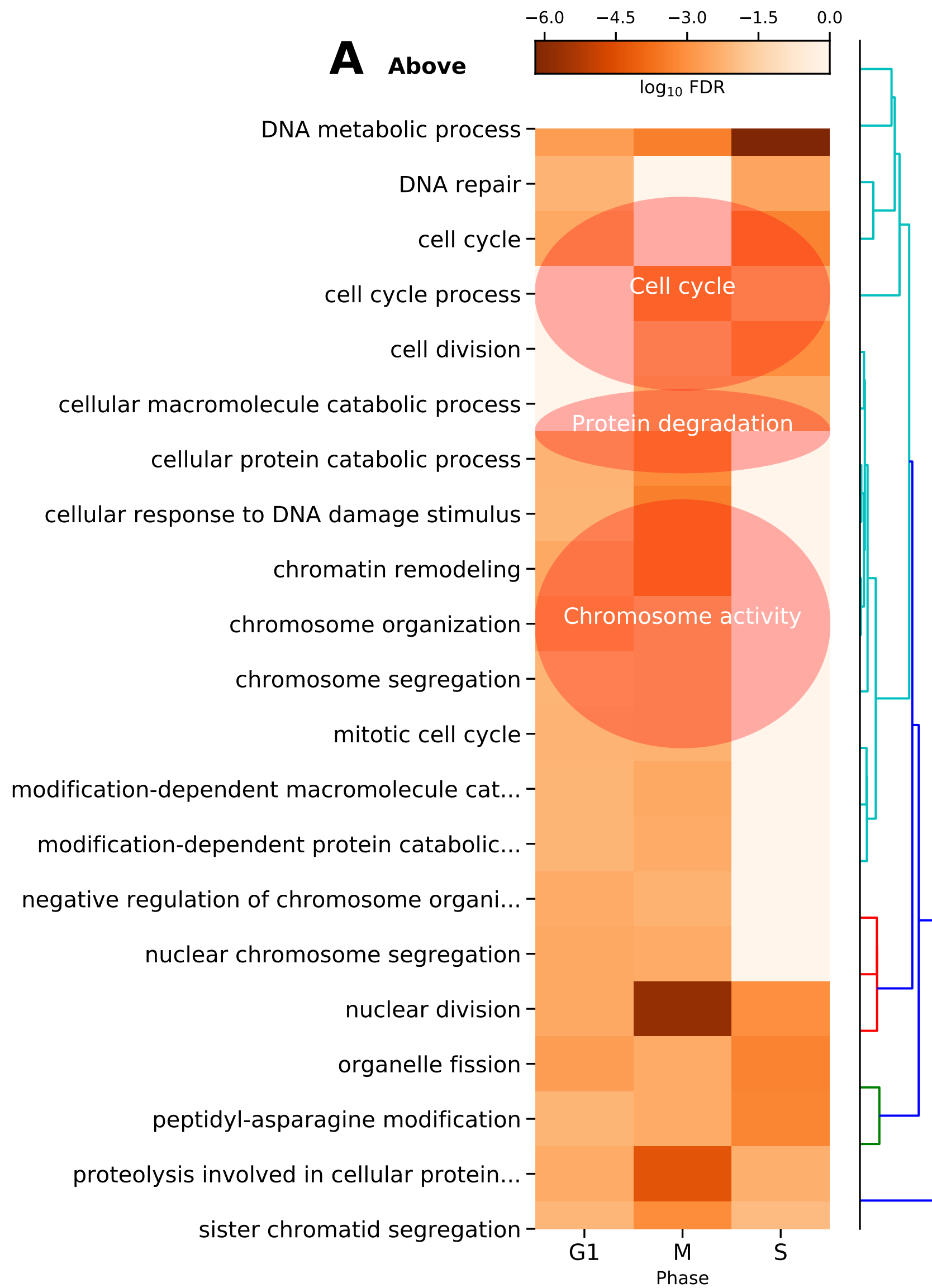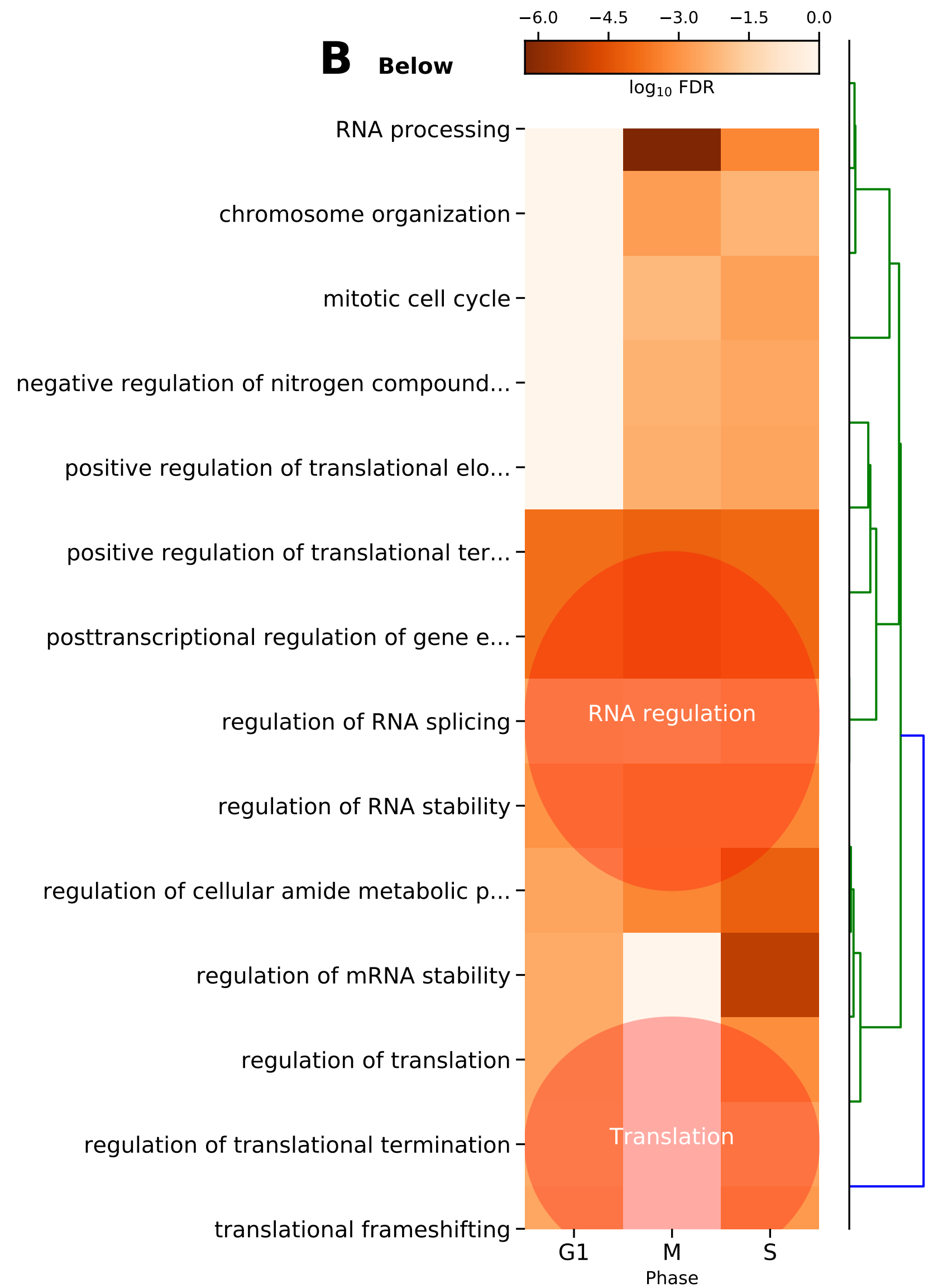

Supplement: Supplementary file 6 — Additional file 6 Hierarchical Clustering of GOBP Terms above (left) and below (right) the regression line (see Fig. 4c). using (log10) p-value FDR with Benjamini correction (p<0.01). Annotated circles (orange) pseudo-group regions of interest for each plot. Dendrograms aside each plot identify grouped-distance. [file 12859_2019_3150_MOESM6_ESM.pdf]

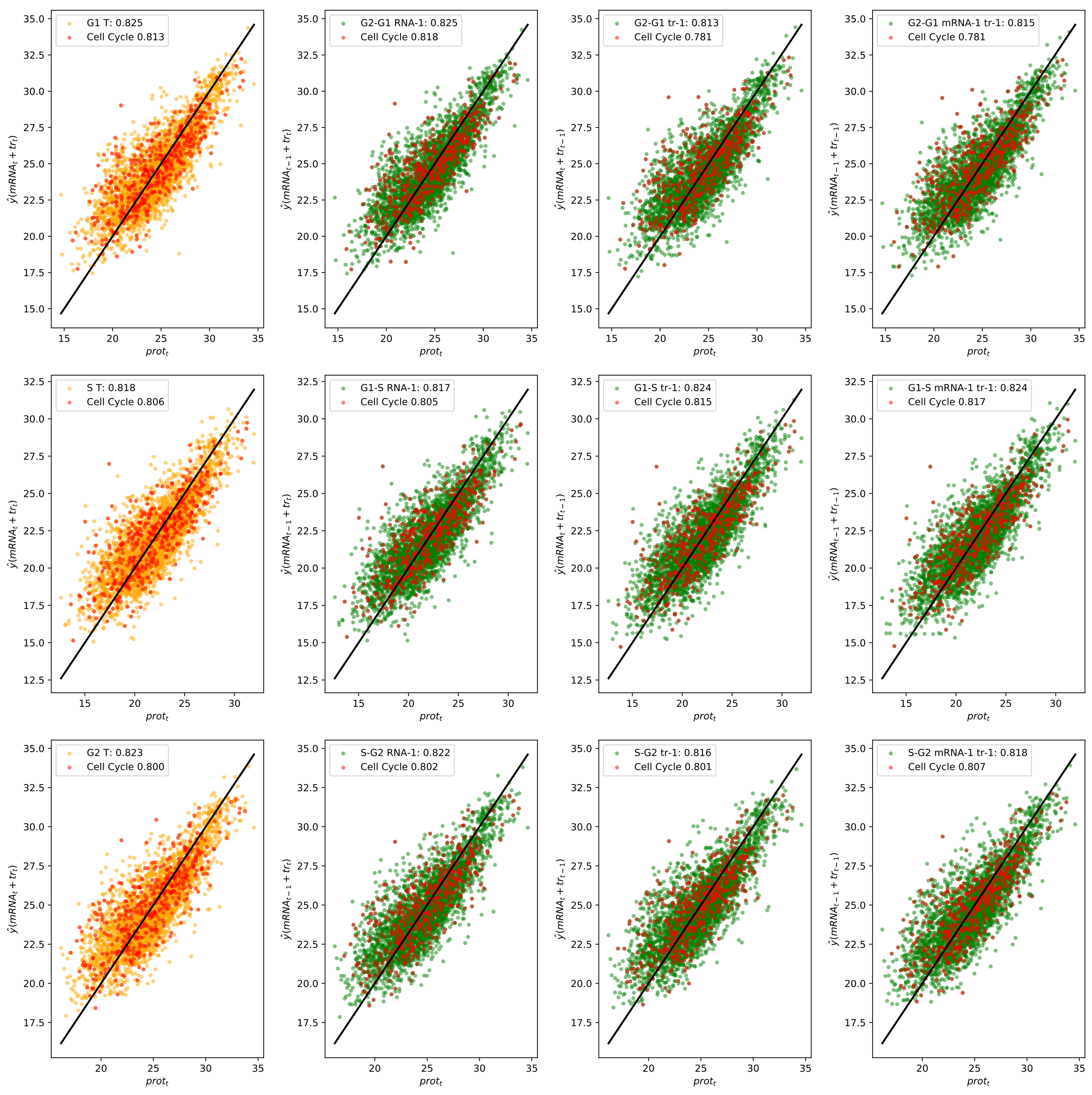

Supplement: Supplementary file 7 — Additional file 7 Scatterplots of protein levels against predicted protein $\hat p$p^ generated from different mRNA/translation measurement inputs. a) mRNAt b) mRNAt−1 c) translationt−1 or d) mRNAt−1,translationt−1. From top: S, G2/M, G1 cell cycle phase. Yellow plots refer to the normal model (see Fig. 4a). Cell cycle terms are annotated for using Gene Ontology and overlaid with correlation. t refers to the cell cycle step (G1, S or G2/M). [file 12859_2019_3150_MOESM7_ESM.pdf]

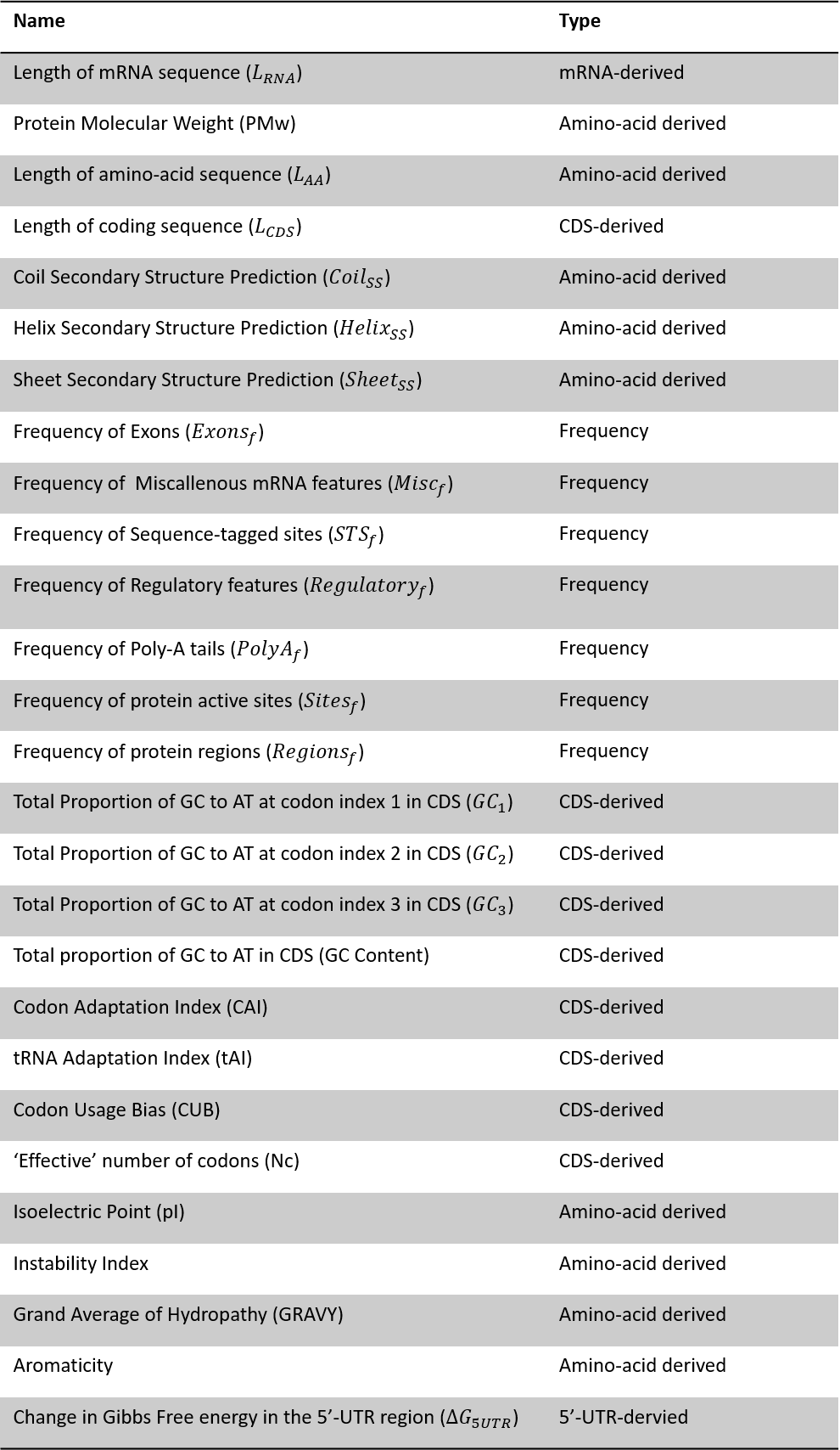

Supplement: Supplementary file 8 — Additional file 8 Table of expanded feature names with abbrievations. Includes input feature abbrievations used in Fig. 2. [file 12859_2019_3150_MOESM8_ESM.tif]
