# Supplementary material for: Uncovering extensive post-translation regulation during human cell cycle progression by integrative multi-’omics analysis
Source: BMC Bioinformatics. 2019 Oct 29;20:536. doi: 10.1186/s12859-019-3150-5 (PMC6820968; doi:10.1186/s12859-019-3150-5)

Feature Selection, Section 3

Our methods of feature selection we will cover are:

1. Recursive Feature Elimination (RFE)
2. L_1 sparsity-inducing regularization (LASSO)
3. Selecting k-Best (ANOVA)

For all of our models using gradient-boosted regression trees (GBRT), these are our tuned parameters from model selection:

1. G1; learning rate = 0.02, max depth = 3, min samples leaf = 10, n_base_estimators = 1000
2. S; learning rate = 0.01, max depth = 3, min samples leaf = 10, n_base_estimators = 1000
3. G2/M; learning rate = 0.02, max depth = 3, min samples leaf = 5, n_base_estimators = 1000

Feature matrix X is always standardised/normalized using $(X - mean(X))/std(X)$, y is not standardized. G2 and M are inter-changeable as labels for the final cell cycle phase.

RFE

Firstly, we combine recursive feature elimination method from scikit-learn (python) with 10-fold cross validation to automatically filter features of interest. We use a gradient-boosted regression tree as the estimator, with 1000 base decision tree estimators, using the negative mean squared error (-MSE) to maximise as the scoring function:

$$MSE = \frac{1}{N} \sum_{i=1}^{N} (y - \hat{y})^2 \tag{1}$$

where $\hat{y}$ is the predicted abundance, y is the measured abundance.

The results of this only drop around 2 features per cell cycle phase, indicating that MSE doesn't seem to improve when features are dropped (Fig 1).

We see a clear a clear threshold area around 7-8 features where -MSE appears to flatten out and not increase, therefore we formulate a method

Figure 1: RFECV fails to select a suitable feature subset.

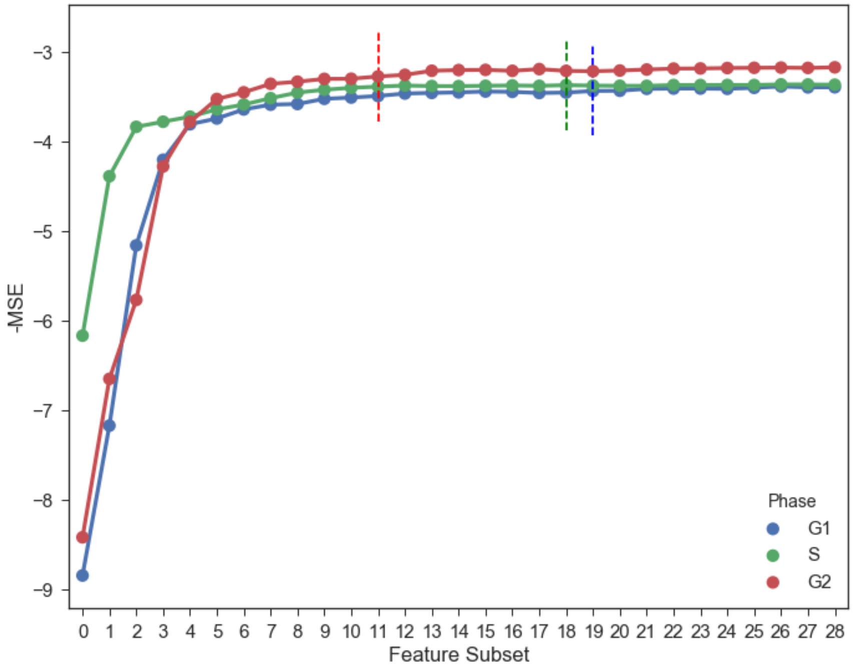

where we choose the number of features to select based on the change in MSE with respect to each subset, which is approximated using euler's method:

$$\frac{dMSE}{dF} \approx (MSE_{n+1} - MSE_n) < \eta \tag{2}$$

where we choose η as a small constant tolerance, where the number of features is where the change begins to slow down in terms of -MSE increase. Here we choose $\eta = 0.005$. Lines are drawn on the graph as to n_features selected ($\hat{p}$) per cell cycle phase, colour-respective.

Using $\hat{p}$, we generate new models of GBRT without CV, indicating the number of features we want the recursion to stop at. This yields a ranking for each of the features, per cell cycle phase, in addition to feature importances for the features that remain (Fig 2).

These selected features are stored and used later on in LOOCV analysis to reduce the feature matrix.

Figure 2: Feature importances generated from RFE-GBRT.

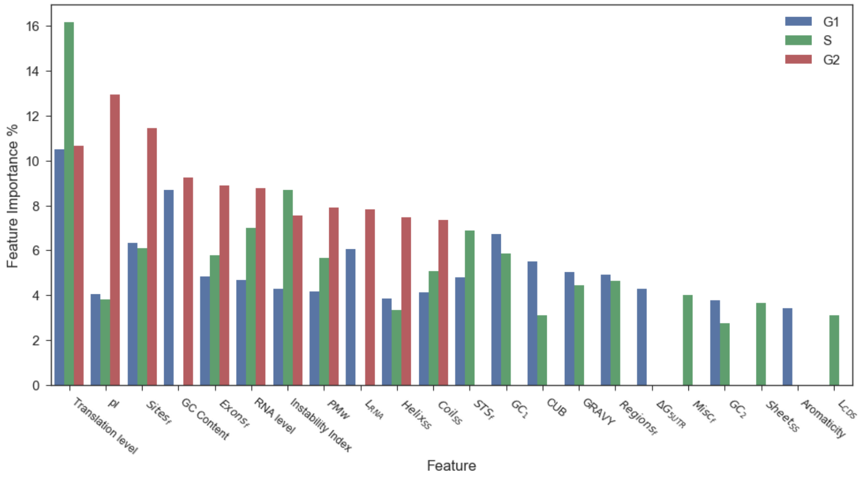

L_1 Regularization

Firstly, to find an optimal α for the regularization term, we use LASSO without GBRT, using a brute-force grid search method, across 10-fold cross validation, with $\alpha \in [10^{-3}, 10^1]$, using the negative mean squared error as before as the scoring function (Equation 1).

We begin to see a drop in accuracy around $\alpha \approx 0.1$, again with no real increase in -MSE at any particular stage within the range.

We then repeat the run by not predicting using the LASSO regressor, but using the sparsity-inducing coefficients to induce sparsity in a reduced X matrix, which is then used as input to a GBRT model. The results of these are shown in Fig 3 in the main paper. For Fig 3A, like the RFE example we choose a threshold η which signifies where the change in MSE should not exceed by, the inverse of described in Equation 2. We chose $\eta = 0.02$ in LASSO case. We then generated 10-fold cross validated test scores (R^2) across a grid of 30 α points, as $\alpha \in [10^{-3}, 1]$, for each cell cycle phase. This generated optimal $\alpha = [0.05, 0.07]$ range. Using these regularizers, we generated final GBRT models using single alpha per cell cycle phase, using 10-fold cross validation and extracting the feature importances from each estimator, and plotting the mean (std as error bars) in Fig 3C.

Figure 3: LASSO only parameter tuning of α versus -MSE. *Error bars $\pm$SD 10-fold CV.*

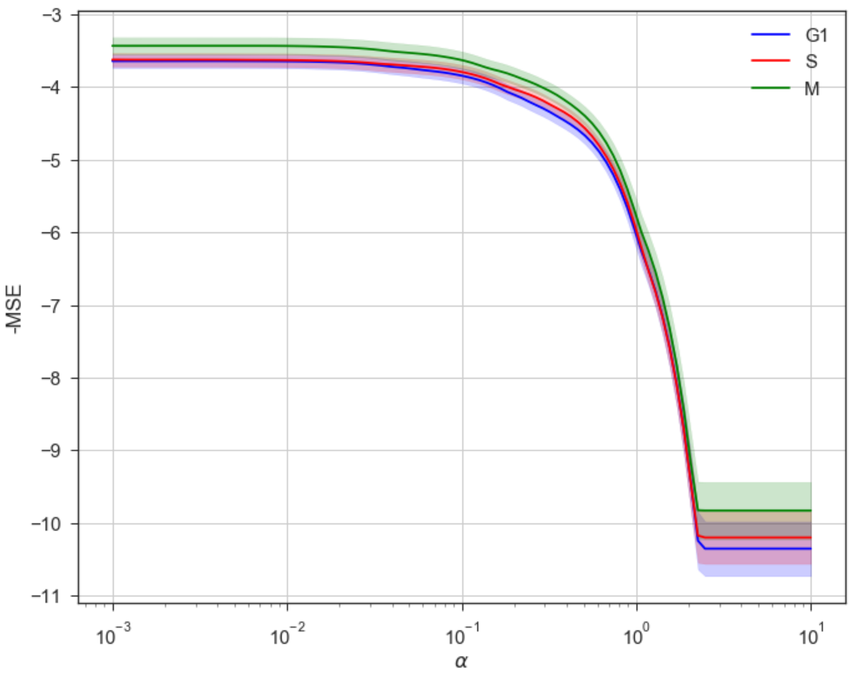

Select K Best

In this feature selector, we optimise to find the most suitable k using Analysis of Variance (ANOVA). This entails, for a given k, calculating the F-value for each feature calculated as the covariance between features, and selecting features with the largest F-value. We create a grid of $k \in [1, P]$, where P is the total number of features initially, then we use a Pipeline object in scikit-learn to reduce X with Selecting the k best features as described above, then using a GBRT model for prediction. Again we use the negative mean-squared error as a scoring function (see Equation 1), with 10-fold cross validation.

We see that unlike the first 2 feature selectors, there is not an exponential curve but rather linear decreases in MSE beyond $k = 3, 4$. Therefore there was no clear threshold to choose to find an optimal k, so we followed the default settings in scikit-learn, which by default select k to be half of the original number of features P. In this case, $k = 14$ for all cell cycle phases.

Figure 4: Parameter tuning of k versus -MSE of resulting model on test data. *Error bars ±SD 10-fold CV.*

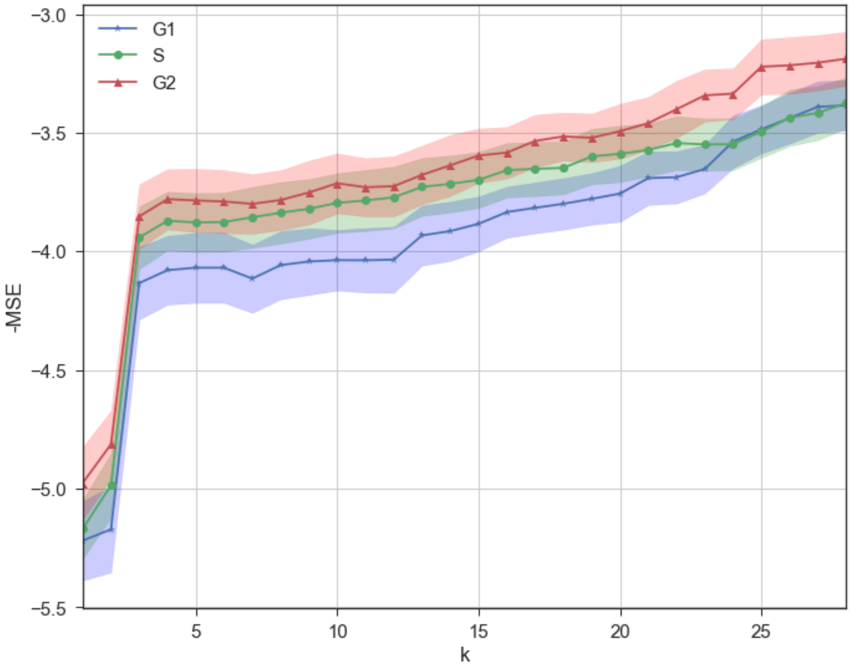

Using optimal k we created a GBRT model with 10-fold cross validation, as with the previous 2 feature selection procedures (Figure 5). Interestingly K Best chooses very different features in order compared to the 2 previous selectors, ranking mRNA level quite high with some mRNA-derived features.

Figure 5: Feature importances generated from KBest-GBRT. *Error bars ±SD 10-fold CV.*

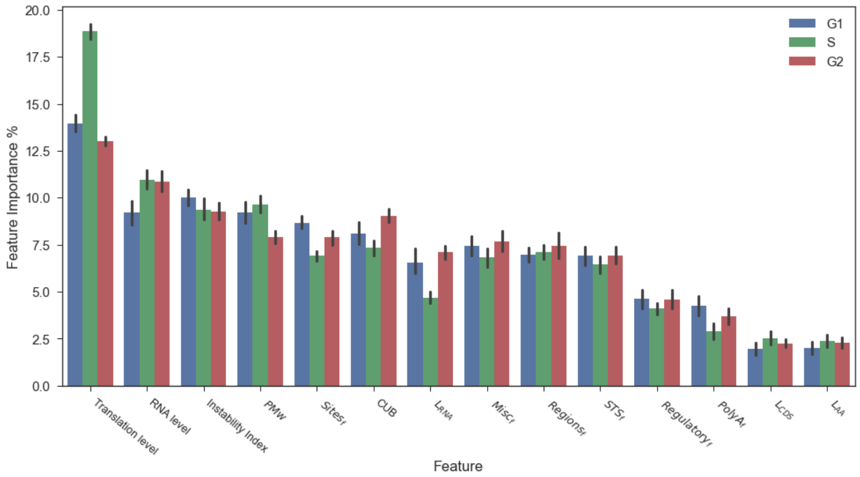

Supplement: Supplementary file 11 — Additional file 11 Feature Selection procedure. Description of the feature selection process for RFE, L1 and Select k-Best, including parameter choices in this pdf. [file 12859_2019_3150_MOESM11_ESM.pdf]
